# Remarkable shift in structural and functional properties of an animal charcoal-polluted soil accentuated by inorganic nutrient amendment

**DOI:** 10.1186/s43141-020-00089-9

**Published:** 2020-11-11

**Authors:** Lateef Babatunde Salam, Oluwafemi Sunday Obayori

**Affiliations:** 1 Department of Biological Sciences, Microbiology unit, Summit University, Offa, Kwara Nigeria; 2 Department of Microbiology, Lagos State University, Ojo, Lagos Nigeria

**Keywords:** Animal charcoal-polluted soil, Carbon-free mineral medium, Hydrocarbon degradation, Illumina shotgun sequencing, Microbial Community Structure, Soil microcosm

## Abstract

**Background:**

Soils polluted with animal charcoal from skin and hide cottage industries harbour extremely toxic and carcinogenic hydrocarbon pollutants and thus require a bio-based eco-friendly strategy for their depuration. The effects of carbon-free mineral medium (CFMM) amendment on hydrocarbon degradation and microbial community structure and function in an animal charcoal-polluted soil was monitored for 6 weeks in field moist microcosms consisting of CFMM-treated soil (FN4) and an untreated control (FN1). Hydrocarbon degradation was monitored using gas chromatography-flame ionization detector (GC-FID), and changes in microbial community structure were monitored using Kraken, while functional annotation of putative open reading frames (ORFs) was done using KEGG KofamKOALA and NCBI’s conserved domain database (CDD).

**Results:**

Gas chromatographic analysis of hydrocarbon fractions revealed the removal of 84.02% and 82.38% aliphatic and 70.09% and 70.14% aromatic fractions in FN4 and FN1 microcosms in 42 days. Shotgun metagenomic analysis of the two metagenomes revealed a remarkable shift in the microbial community structure. In the FN4 metagenome, 92.97% of the population belong to the phylum *Firmicutes* and its dominant representative genera *Anoxybacillus* (64.58%), *Bacillus* (21.47%) and *Solibacillus* (2.39%). In untreated FN1 metagenome, the phyla *Proteobacteria* (56.12%), *Actinobacteria* (23.79%) and *Firmicutes* (11.20%), and the genera *Xanthobacter* (9.73%), *Rhizobium* (7.49%) and *Corynebacterium* (7.35%), were preponderant. Functional annotation of putative ORFs from the two metagenomes revealed the detection of degradation genes for aromatic hydrocarbons, benzoate, xylene, chlorocyclohexane/chlorobenzene, toluene and several others in FN1 metagenome. In the FN4 metagenome, only seven hydrocarbon degradation genes were detected.

**Conclusion:**

This study revealed that though CFMM amendment slightly increases the rate of hydrocarbon degradation, it negatively impacts the structural and functional properties of the animal charcoal-polluted soil. It also revealed that intrinsic bioremediation of the polluted soil could be enhanced via addition of water and aeration.

**Supplementary Information:**

The online version contains supplementary material available at 10.1186/s43141-020-00089-9.

## Background

The Nigerian environment is dotted by tens of thousands of sites polluted with animal charcoal. These are sites where animal hides, mainly from cow, are burnt or singed to remove the fur in the process of producing local delicacies called ‘ponmo’.

The process involved often includes the use of kerosene, diesel, spent engine oil, plastics and old tyre as fuel [1]. It is poorly regulated and characterized by indiscriminate disposal of wastes, which are rich in animal charcoal laced with various types of hydrocarbons, including aliphatics, aromatics, polycyclic aromatic hydrocarbons (PAHs), dioxins, furans, benzenes and heavy metals [2, 3]. These wastes eventually find their way into surrounding soils, underground waters, runoffs and surface waters. Recent report from a study of such site revealed hydrocarbon concentration far in excess of regulatory limit but very low concentration of inorganic nutrient [3, 4]. Since these sites are usually located around abattoirs and water canals or small streams, it is doubtless that this process could pose serious hazards to the ecosystem [5].

Hydrocarbons and heavy metal pollutants are known to pose health hazards resulting from toxicity, mutagenicity, teratogenicity and carcinogenicity [6, 7]. Although remediation efforts in the past focused more on physical and chemical approaches which are often very rapid, in the last few decades, attention has been directed to bioremediation or application of biological processes. This is mainly due to inherent shortcomings of the traditional processes and the benign, effective, environmentally-friendly and relatively inexpensive attributes of bioremediation approach [8]. In order to be able to implement an effective bioremediation programme, there is need for information on the pre-remedial physicochemistry and community diversity of the polluted system as well as monitoring of the same during the bioremediation campaign [9, 10].

Furthermore, whereas there is a plethora of reports on the characteristics of hydrocarbon-polluted sites in Nigeria, there is presently a dearth of information on the physicochemistry and microbial community diversity of majority of the animal charcoal-polluted sites across the length and breadth of the country. Similarly, despite the presence of toxic and carcinogenic hydrocarbon pollutants in animal charcoal-polluted sites, there is paucity of efforts aimed at effective bioremediation of these sites. Availability of limiting nutrients such as phosphorus and nitrogen are pivotal for effective bioremediation of polluted sites. Biostimulation via addition of these limiting nutrients (organic or inorganic) has demonstrably enhanced hydrocarbon degradation [9, 10] While carbon-free mineral medium (CFMM), rich in nitrogen and phosphorus sources, have been used as an enrichment medium for isolation of hydrocarbon degraders [11], its use as a biostimulant to enhance hydrocarbon degradation in soil has not been established.

Culture-dependent approaches have been used to assess microbial community structure of polluted environments. However, the realization that it only reveals < 1% of the members of the community makes it less desirable for microbial ecology studies [12]. The use of a culture-independent approach such as shotgun metagenomics premised on sequencing the entire metagenome not only providing deep insights on the microbial ecology of contaminated sites but also giving a snapshot of metabolic properties of the members of the community [13, 14].

Here we report the dynamics of microbial community diversity and metabolic properties of a microcosm from a ‘ponmo’ cottage industry soil treated with carbon-free mineral medium (CFMM). This is with a view to determining the efficiency of nutrient addition in reclamation and detection of novel genes and functionalities associated with animal charcoal contaminated systems. To the best of knowledge, this is the first report of shotgun metagenomic profiling of such sites.

## Methods

### Sampling site description and soil microcosm setup

Composite animal charcoal-polluted soil samples were collected at an abattoir located in Ilorin, Kwara State, Nigeria. The sampling site, which has been in existence for 12 years, is where slaughtered animal hides and skin were burnt to remove the furs to produce a local Nigerian delicacy, ‘ponmo’. The coordinates of the sampling site were latitude 8.471498 N and longitude 4.531245 E. Soil samples were collected at a depth of 10–12 cm with a sterile hand trowel after clearing debris from the soil surface. It was sieved using a 2-mm mesh size sieve. The sieved soil was thoroughly mixed in a large plastic bag to ensure homogeneity and was used without air-drying. The protocols for the microcosm setup is as described by [10] with slight modifications. Sieved soil (1 kg) measured and placed in an open pan was designated FN1. The second soil microcosm designated FN4 contain 1 kg of sieved soil amended with 100 ml carbon-free mineral medium (CFMM; g L^−1^: NH_4_NO_3_, 3.0 g; Na_2_HPO_4_, 2.2 g; KH_2_PO_4_,0.8 g; MgSO4·7H_2_O, 0.1 g; FeCl_3_·6H_2_O, 0.05 g; and CaCl_2_·2H_2_O, 0.05 g; pH 7.0). The setups (in triplicates) were incubated at room temperature (25 ± 3 °C) for 6 weeks and flooded with 100 ml sterile distilled water to maintain a moisture level of 25%.

The physicochemistry and heavy metal content of the polluted soil were determined as described previously [3, 15]. The physicochemical properties indicated a pH of 5.51 ± 0.02, moisture content of 8.59% (± 0.16) and total organic content of 18.99 ± 0.18%. The nitrogen, phosphorus and potassium contents are 5.99 ± 0.06, 5.55 ± 0.12 and 12.99 ± 0.04 mg kg^−1^ soil, respectively. Similarly, background heavy metal content of the polluted soil revealed in mg kg^−1^ soil, the presence of iron (72.41 ± 0.72), lead (3.22 ± 0.11), zinc (12.50 ± 0.42, copper (8.99 ± 0.32), manganese (3.05 ± 0.01), cadmium (0.72 ± 0 .01), nickel (4.01 ± 0.01) and chromium (2.89 ± 0.02), respectively. Samples were taken from FN1 and FN4 microcosms for hydrocarbon content analyses at days 0, 21 and 42, respectively.

### Hydrocarbon content analysis of the microcosms

Hydrocarbon content of the soil samples was determined by first drying the polluted soil samples (10 g) with 10 g anhydrous Na_2_SO_4_ in an extraction thimble. A mixture of analytical grade dichloromethane and acetone (10 ml; 1:1, v/v) was thereafter added and shaken for 30 min in a mechanical shaker. Collected samples were filtered into a glass beaker using a glass wool plugged into a glass funnel with 1 g anhydrous Na_2_SO4. The extraction was twice repeated and concentrated to 10 ml at 60 °C using a rotary evaporator, after which 10 ml of hexane was added and further concentrated to about 1 ml at 60 °C. Clean-up and fractionation of the extract was done using silica gel permeation chromatography. Mixture of hexane and acetone (1:3 v/v; 10 ml) was used to extract the aliphatic fraction while 10 ml of *n*-hexane was used to extract the aromatic fraction. Residual aliphatic and aromatic hydrocarbon fractions were determined by gas chromatography equipped with flame ionization detector (GC-FID) using an OV®-3 column. The carrier gas is nitrogen. The injector and detector temperatures were maintained at 220 °C and 270 °C, respectively. The column was programmed at an initial temperature of 50 °C for 2 min, ramped at 10 °C/min to 250 °C and held for 5 min. The air flow, hydrogen flow and nitrogen flow rates are 450, 45 and 22 ml min^−1^, respectively.

### Total DNA extraction, shotgun metagenomics, processing of raw fastq reads and read-based classification

Total DNA used for metagenomic analysis was extracted directly from the two soil microcosms, FN1 and FN4. To discern the microbial community structure of the animal charcoal-polluted soil prior to CFMM amendment, total DNA was extracted from the soil (FN1) immediately after sampling. For metagenomic evaluation of the effects of CFMM addition (100 ml kg^−1^ of soil) on the microbial community of the animal charcoal-polluted soil, the total DNA was extracted from FN4 microcosm 6 weeks post CFMM addition. Total DNA were extracted from the sieved soil samples (0.25 g) using ZYMO soil DNA extraction kit (Model D 6001, Zymo Research, USA) following the manufacturer’s instructions. The quality and concentration of the extracted total DNA was ascertained using a NanoDrop spectrophotometer and electrophoresed on a 0.9% (w/v) agarose gel, respectively. Shotgun metagenomics of FN1 and FN4 microcosms was prepared using the Illumina Nextera XT sample processing kit and sequenced on a MiSeq. The protocols for total DNA preparation for Illumina shotgun sequencing were as described previously [10, 16].

Pre-processing of fastq raw reads for quality profiling, read filtering, adapter trimming, quality filtering, polyG/polyX tail trimming and per-read quality pruning was carried out using fastp, an ultra-fast FASTQ preprocessor [17]. The processed raw fastq reads was submitted to the Kraken taxonomic sequence classifier database [18] for read-based classification.

### Functional annotation of metagenomics reads

Sequence reads generated from each of the metagenome were assembled individually using the make.contigs command in the MOTHUR metagenomic analysis suite [19]. Gene calling was performed on the assembled FN1 and FN4 contigs using MetaGene [20] to predict open reading frames (ORFs). The predicted genes (ORFs) were functionally annotated using the KEGG KofamOALA [21], which assigns K numbers to the predicted genes by HMMER/HMMSEARCH against KOfam (a customized HMM database of KEGG Orthologs), and the NCBI’s conserved domain database CDSEARCH/cdd v 3.15 (CDD) [22]. Taxonomic affiliation of the putative genes in the metagenomes was determined using AAI Profiler, which calculate average amino acid identity (AAI) between a query proteome (ORFs) and all target species in the Uniprot database [23].

### Accession number

The data, metadata and sequence reads of the FN1 and FN4 metagenomes used in this study have been deposited in the European Nucleotide Archive (ENA) at EMBL-EBI under the accession number PRJEB37880 https://www.ebi.ac.uk/ena/data/view/PRJEB37880

### Statistical analyses

Statistical Analysis of Metagenomic Profiles, version 2 (STAMP) software [24] was used to statistically analyse the distinct taxonomic levels for each of the metagenomes retrieved from Kraken. Two-sided Fisher’s exact test with Newcombe–Wilson confidence interval method was used to determine the significance of the relative proportion difference in taxonomic distribution of the FN1 and FN4 metagenomes, while Benjamini–Hochberg FDR was applied for correction. Unclassified reads were not used for analyses, and results with *q* < 0.05 were considered significant. The biological relevance of the statistic taxa was evaluated by applying a difference between the proportions of at least 1% and a twofold ratio between the proportions.

## Results

### Kinetics of hydrocarbon degradation in FN1 and FN4 soil microcosms

The degradation of aliphatic and aromatic hydrocarbons (HC) in FN1 and FN4 soil microcosms was monitored using GC/FID (Figure S1 and S2). In FN1 microcosm, the residual aliphatic HC content (554.98 mg/kg; 100%) decreased to 73.07% (405.51 mg/kg) after 21 days, corresponding to removal of 26.93% (149.47 mg/kg). At the end of 42 days, further decrease to 29.91% (165.98 mg/kg) in the residual aliphatic HC was observed, corresponding to removal of 70.09% (389 mg/kg) aliphatic HC (Figure S1). The residual aromatic HC content (471.56 mg/kg; 100%) decreased to 67.26% (317.19 mg/kg) after 21 days corresponding to the removal of 32.74% (154.37 mg/kg). The residual aromatic HC content decreased further at the end of 42 days, to 29.86% (140.81 mg/kg), corresponding to the removal of 70.14% (330.75 mg/kg) aromatic HC (Figure S2).

In FN4 microcosm, the residual aliphatic HC content (554.98 mg/kg; 100%) decreased to 51.27% (284.53 mg/kg) after 21 days, corresponding to removal of 48.73% (270.45 mg/kg). Further decrease in the residual aliphatic HC to 15.98% (88.66 mg/kg) was observed at the end of 42 days, corresponding to the removal of 84.02% (466.32 mg/kg) aliphatic HC (Figure S3). The residual aromatic HC content (471.56 mg/kg; 100%) decreased to 45.80% (215.98 mg/kg) after 21 days corresponding to the removal of 54.20% (255.58 mg/kg). Further decrease in the residual aromatic HC content to 17.62% (83.11 mg/kg) was observed after 42 days, corresponding to the removal of 82.38% (388.45 mg/kg) aromatic HC (Figure S4).

Significant changes in the degradation pattern of the hydrocarbon fractions were observed in FN1 and FN4 microcosms as shown in the GC fingerprints (Figures S1 and S2) and Table 1. In FN1 microcosm, the GC fingerprints of the aliphatic fractions showed complete disappearance of ethane, propane, cyclopropane, butane, methylpropane, pentane, methylbutane and tricosane fractions at the end of 42 days. Significant reductions of some fractions to < 15% (hexane, octane, 2,2,4-trimethylpentane, decane, dodecane) and < 30% (nonadecane, eicosane, docosane) were also observed. The GC fingerprint of the aromatic fractions revealed the disappearance of indeno(123-cd)pyrene fractions, and significant reduction to < 15% of fluoranthene, benzo(a)pyrene and dibenzo(a)anthracene fractions, respectively (Figure S1, Table 1).

**Table 1:** Percentage representative aliphatic and aromatic hydrocarbon fractions remaining in the FN1 and CFMM-amended FN4 soil microcosms after 21 and 42 days of incubation at room temperature. Values were calculated from peak areas on day 21 and day 42, respectively, relative to peak area values for day 0

Hydrocarbon fractions	Day 0	Day 21	Day 42	Day 0	Day 21	Day 42
**Aliphatic fractions**	**FN1**			**FN4**		
Ethane	100	0.00	0.00	100	18.95	0.00
Propane	100	0.00	0.00	100	0.00	0.00
Cyclopropane	100	0.00	0.00	100	0.00	0.00
Butane	100	0.00	0.00	100	0.00	0.00
Methylpropane	100	0.00	0.00	100	0.00	0.00
Pentane	100	0.55	0.00	100	0.55	0.00
Methylbutane	100	4.23	0.00	100	4.23	0.00
Hexane	100	6.52	9.81	100	3.04	0.00
Heptane	100	83.98	63.51	100	65.31	21.45
Octane	100	12.68	5.22	100	9.17	0.00
2,2,4-Trimethylpentane	100	39.36	14.95	100	33.64	7.38
Decane	100	68.08	13.95	100	54.90	4.71
Dodecane	100	53.28	12.83	100	34.55	9.11
Tetradecane	100	77.92	43.51	100	82.61	34.06
Hexadecane	100	76.73	37.93	100	53.13	10.78
Heptadecane	100	77.73	39.49	100	48.90	26.58
Pristane	100	88.29	52.22	100	46.17	21.20
Octadecane	100	Acc.	33.72	100	65.35	20.38
Phytane	100	73.30	32.67	100	49.19	18.25
Nonadecane	100	60.07	20.07	100	29.70	15.62
Eicosane	100	31.32	28.27	100	34.36	23.86
Docosane	100	50.99	20.57	100	35.96	22.41
Tricosane	100	0.00	0.00	100	0.00	0.00
**Aromatic fractions**						
Naphthalene	100	77.09	68.53	100	77.09	0.00
Acenaphthylene	100	76.37	76.68	100	76.69	76.68
Acenaphthene	100	89.23	89.23	100	89.23	89.23
Fluorene	100	63.57	56.07	100	56.07	56.07
Phenanthrene	100	25.45	20.03	100	21.21	15.05
Fluoranthene	100	11.65	4.26	100	4.26	0.00
Pyrene	100	61.89	36.47	100	46.64	23.81
Benzo(a)anthracene	100	Acc.	55.13	100	80.48	39.17
Chrysene	100	Acc.	68.55	100	Acc.	24.28
Benzo(b)fluoranthene	100	Acc.	34.45	100	79.78	15.03
Benzo(a)pyrene	100	24.18	4.96	100	15.83	0.00
Dibenzo(a)anthracene	100	53.41	10.79	100	35.83	5.95
Benzo(ghi)perylene	100	Acc.	28.00	100	45.07	12.46
Indeno(123-cd)pyrene	100	5.79	0.00	100	2.80	0.00

*Acc* Accumulated, i.e. the concentration of the fraction is higher than day 0 value

In FN4 microcosm, the GC fingerprints of the aliphatic fractions revealed complete disappearance of ethane, propane, cyclopropane, butane, methylpropane, pentane, methylbutane, hexane, octane and tricosane fractions at the end of 42 days. Apart from tetradecane, all the other aliphatic fractions were significantly reduced to < 10% (2,2,4-trimethylpentane, decane, dodecane) and < 30% (heptane, hexadecane, heptadecane, pristane, octadecane, phytane, nonadecane, eicosane, docosane) of their initial concentrations. The GC fingerprints of the aromatic fractions revealed complete disappearance of naphthalene, fluoranthene, benzo(a)pyrene and indeno(123-cd)pyrene fractions at the end of 42 days. Substantial reduction of some aromatic fractions to < 10% (dibenzo(a)anthracene), < 20% (phenanthrene, benzo(b)fluoranthene, benzo(ghi)perylene) and < 30% (pyrene, chrysene) at the end of 42 days was also observed (Figure S2, Table 1).

### General characteristics of the metagenomes

Illumina miseq shotgun sequencing of the two metagenomes resulted in 14,232 and 22,992 sequence reads for FN1 and FN4 with a total of 4,256,742 and 6,878,221 bp, a mean sequence length of 299 bp for both metagenomes, and a mean GC content of 57.31 and 57.72%, respectively. After pre-processing step with fastp, the sequence reads in FN1 and FN4 reduced to 13,500 and 21,624 with a total of 4,035,631 and 6,465,448 bp, the same mean sequence length and GC content of 57.22% for both metagenomes. The duplication rates in FN1 and FN4 sequence reads were 5.1 and 19.3%, while the insert size peak was 468 and 209, respectively. Additional information on the two metagenomes is available at https://www.ebi.ac.uk/ena/data/view/PRJEB37880

### Structural diversity of the metagenomes

**Fig. 1:**
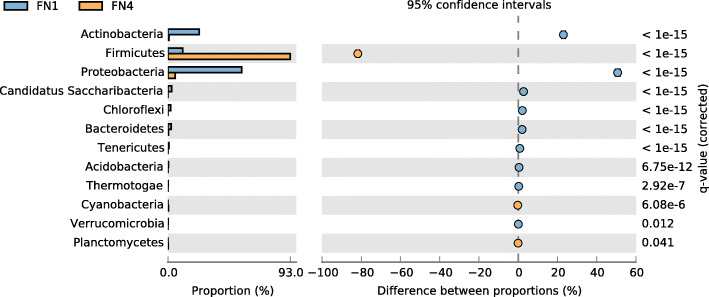
Comparative taxonomic profile of the FN1 and FN4 metagenomes at the phylum level, computed by Kraken. Only phyla with significant biological differences as determined by STAMP (*P* < 0.05, difference between the proportions > 1% and twofold of ratio between the proportions) are shown

Analysis of the microbial community structure of the two metagenomes, FN1 and FN4, revealed significant differences in the taxonomic profiles generated by Kraken. In phylum classification where 14 and 8 phyla were recovered in the FN1 and FN4 metagenomes, the predominant phyla in FN1 are *Proteobacteria* (56.12%), *Actinobacteria* (23.79%) and *Firmicutes* (11.20%). In contrast, the most dominant phylum in the CFMM-amended FN4 metagenome was the *Firmicutes* with 92.97%. Other phyla with reasonable representation in FN4 were *Proteobacteria* (5.47%) and *Actinobacteria* (0.77%). Nine phyla, which are represented in FN1 metagenome, completely disappeared in FN4, while the phyla *Planctomycetes*, *Cyanobacteria* and *Ignavibacteriae*, not represented in FN1, were duly detected in FN4 (Fig. 1).

**Fig. 2:**
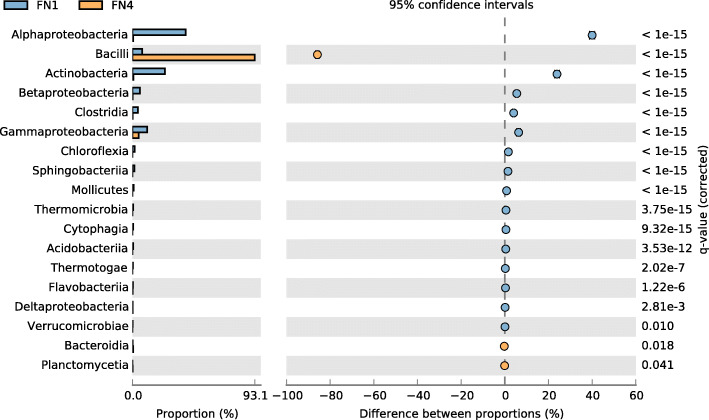
Comparative taxonomic profile of the FN1 and FN4 metagenomes at class delineation, computed by Kraken. Only classes with significant biological differences as determined by STAMP (*P* < 0.05, difference between the proportions > 1% and twofold of ratio between the proportions) are shown

In class delineation, 22 and 11 classes were recovered from the FN1 and FN4 metagenomes. The classes *Alphaproteobacteria* (40.61%), *Actinobacteria* (24.69%) and *Gammaproteobacteria* (11.08%) were preponderant in FN1, while *Bacilli* (93.11%) massively dominate in FN4, along with reasonable representation from the classes *Gammaproteobacteria* (4.74%) and *Actinobacteria* (0.77%). Thirteen classes, which were duly represented in FN1, completely disappeared in FN4, while the classes *Planctomycetia* and *Ignavibacteria* hitherto not present in FN1 were detected in FN4 (Fig. 2).

Order classification revealed 53 and 28 orders in the FN1 and FN4 metagenomes. The predominant orders in FN1 were *Rhizobiales* (26.30%), *Corynebacteriales* (11.06%) and *Propionibacteriales* (6.47%), while *Bacillales* (89.56%), *Enterobacteriales* (3.52%) and *Lactobacillales* (3.45%) were preponderant in FN4. Thirty orders, previously detected in FN1, completely disappeared in FN4 while the orders *Pleurocapsales*, *Planctomycetales*, *Ignavibacteriales*, *Desulfovibrionales* and *Oscillatoriales* hitherto missing in FN1 were duly represented in FN4 (Figure S5).

Family delineation of the FN1 and FN4 metagenomes revealed 99 and 54 families. The dominant families in FN1 were *Xanthobacteraceae* (10.94%), *Rhizobiaceae* (7.97%) and *Corynebacteriaceae* (7.59%), while in FN4, *Bacillaceae* (87.03%), *Enterobacteriaceae* (3.56%) and *Planococcaceae* (2.30%) were preponderant. Fifty-eight families previously detected in FN1 completely disappeared in FN4, while 13 families not detected in FN1 were duly represented in FN4 (Figure S6).

**Fig. 3:**
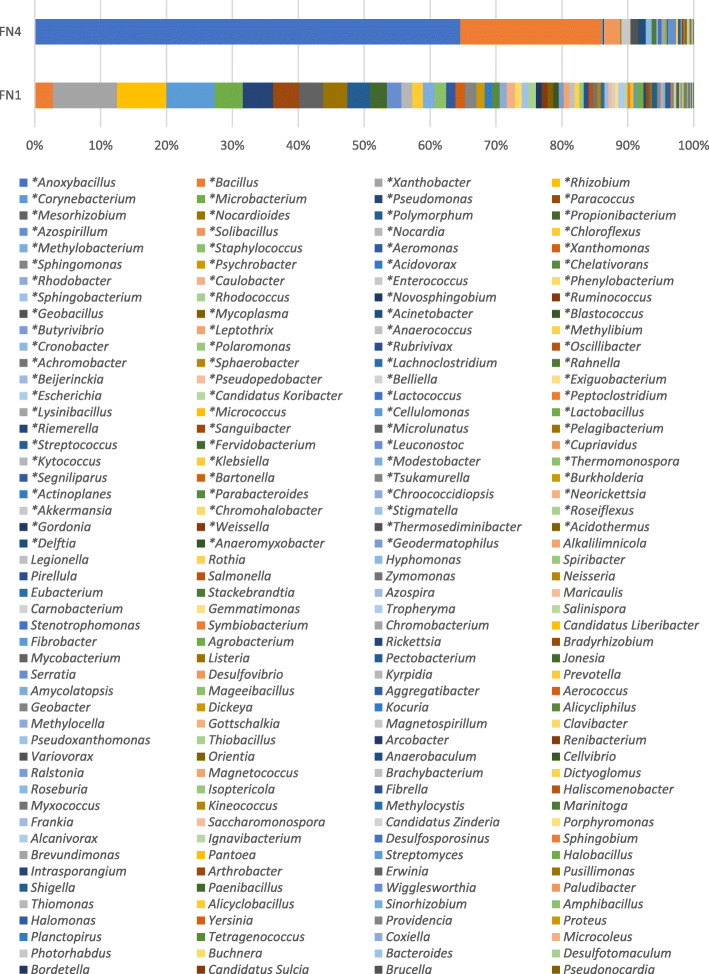
Comparative taxonomic profile of the FN1 and FN4 metagenomes at genus delineation, computed by Kraken. All the genera detected in both metagenomes are shown. *Significant differences between FN1 and FN4 microcosms as determined by STAMP (*P* < 0.05, difference between the proportions > 1% and twofold of ratio between the proportions, as determined by STAMP)

In genus classification, 155 and 95 genera were recovered from the FN1 and FN4 metagenomes. The genera with the highest representation in FN1 metagenome are *Xanthobacter* (9.73%), *Rhizobium* (7.49%) and *Corynebacterium* (7.35%). In the CFMM-amended FN4 metagenome, *Anoxybacillus* (64.58%), *Bacillus* (21.47%) and *Solibacillus* (2.39%) were preponderant. One hundred and one (101) genera previously detected in FN1 metagenome completely disappeared in FN4, while 41 genera, hitherto not detected in FN1, were duly represented in the FN4 metagenome (Fig. 3).

### Functional characterization of the metagenomes

**Fig. 4:**
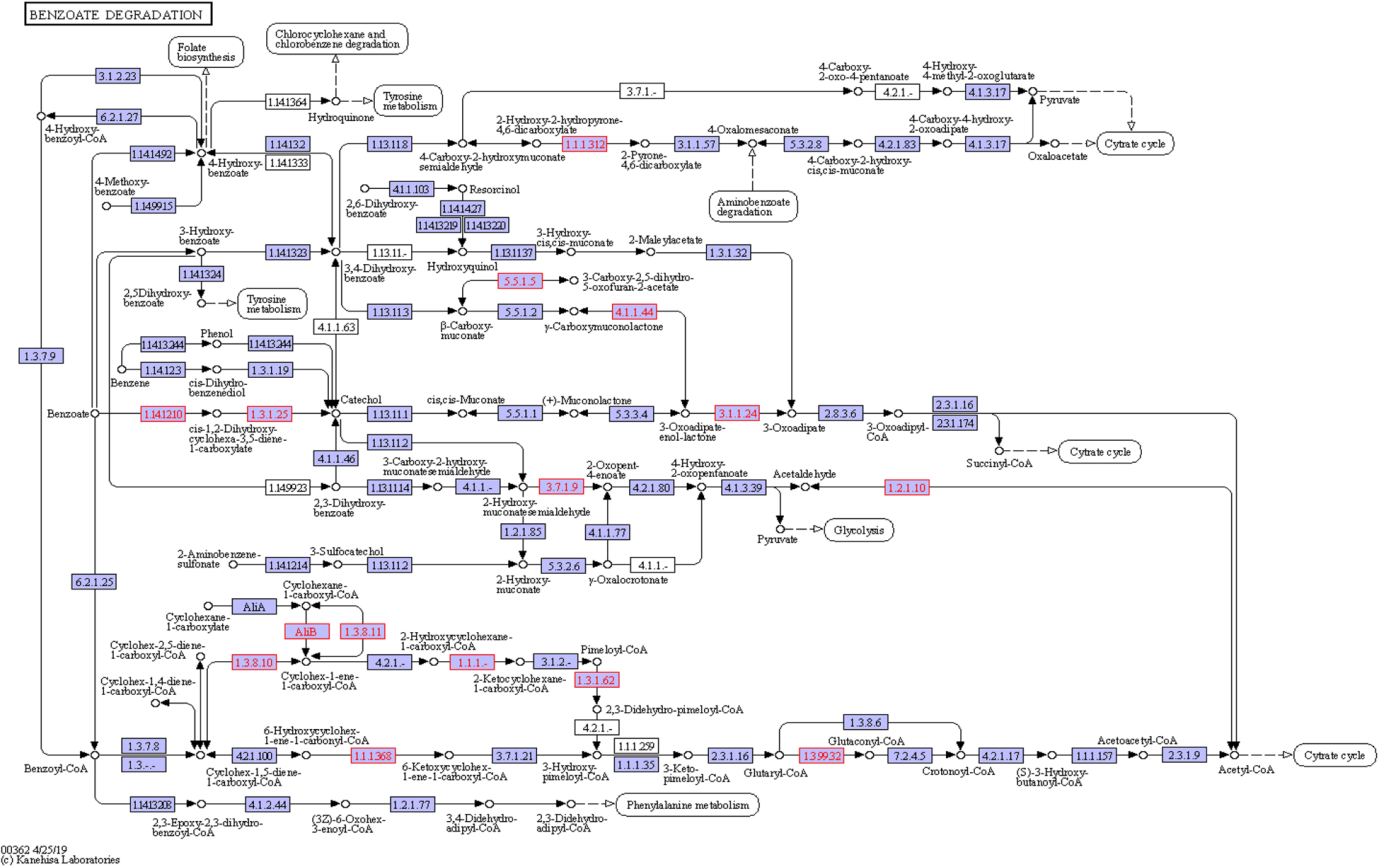
The benzoate degradation pathway performed using the KEGG pathway in KEGG Orthology. EC numbers in red are the benzoate degradation genes identified in the FN1 microcosm. The identified genes include *pcaD*; 3-oxoadipate enol-lactonase [EC:3.1.1.24], mhpF; acetaldehyde dehydrogenase [EC:1.2.1.10], *aliB*; cyclohexanecarboxyl-CoA dehydrogenase [EC:1.3.99.-], pimeloyl-CoA dehydrogenase [EC:1.3.1.62], *benD-xylL*; dihydroxycyclohexadiene carboxylate dehydrogenase [EC:1.3.1.25 1.3.1.-], *benC-xylZ*; benzoate/toluate 1,2-dioxygenase reductase component [EC:1.18.1.-], *badH*; 2-hydroxycyclohexanecarboxyl-CoA dehydrogenase [EC:1.1.1.-], *had*; 6-hydroxycyclohex-1-ene-1-carbonyl-CoA dehydrogenase [EC:1.1.1.368], *dmpD*; 2-hydroxymuconate-semialdehyde hydrolase [EC:3.7.1.9], *ligC*; 2-hydroxy-4-carboxymuconate semialdehyde hemiacetal dehydrogenase [EC:1.1.1.312], *CMLE*; carboxy-cis,cis-muconate cyclase [EC:5.5.1.5], *pcaL*; 3-oxoadipate enol-lactonase / 4-carboxymuconolactone decarboxylase [EC:3.1.1.24 4.1.1.44], *acd*; glutaryl-CoA dehydrogenase (non-decarboxylating) [EC:1.3.99.32], cyclohex-1-ene-1-carbonyl-CoA dehydrogenase [EC:1.3.8.10] and cyclohexane-1-carbonyl-CoA dehydrogenase [EC:1.3.8.11]

**Fig. 5:**
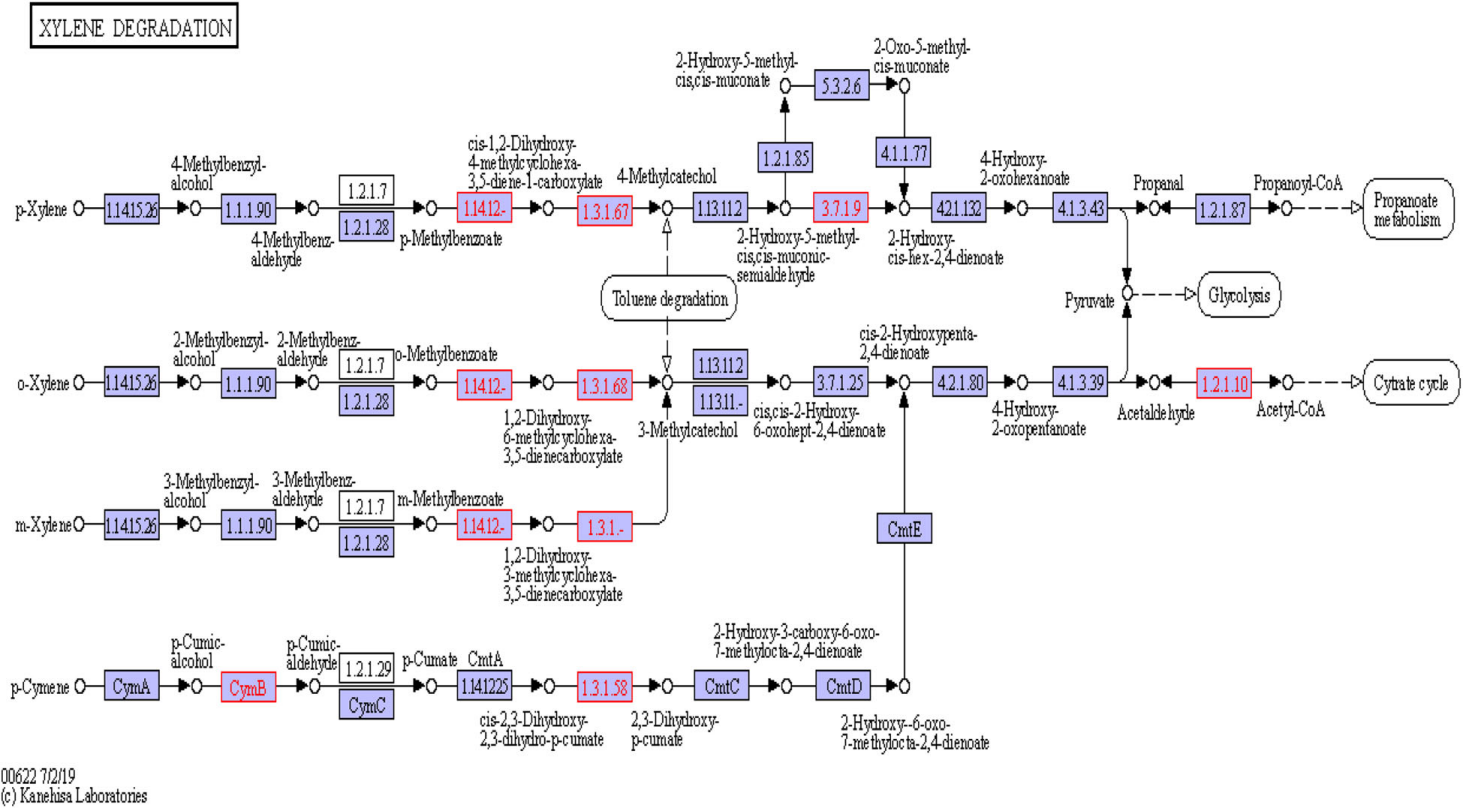
The xylene degradation pathway performed using the KEGG pathway in KEGG Orthology. EC numbers in red are the xylene degradation genes identified in the FN1 microcosm. The identified genes are *mhpF*; acetaldehyde dehydrogenase [EC:1.2.1.10], *benD-xylL*; dihydroxycyclohexadiene carboxylate dehydrogenase [EC:1.3.1.25 1.3.1.-], *benC-xylZ*; benzoate/toluate 1,2-dioxygenase reductase component [EC:1.18.1.-], *dmpD*; 2-hydroxymuconate-semialdehyde hydrolase [EC:3.7.1.9], *cymB*; p-cumic alcohol dehydrogenase and *cmtB*; 2,3-dihydroxy-2,3-dihydro-p-cumate dehydrogenase [EC:1.3.1.58]

Diverse hydrocarbon degradation genes were detected in FN1 metagenome as shown in Table 2. Putative genes responsible for degradation of benzoate (*pcaD*, *mhpF*, *aliB*, *benD-xylL*, *benC-xylZ*, *badH*, *had*, *dmpD*, *ligC*, *CMLE*, *pcaL*, *acd*, among others), xylene (*mhpF*, *benD-xylL*, *benC-xylZ*, *dmpD*, *cymB*, *cmtB*), chlorocyclohexane/chlorobenzene (*dehH*, *dhaA*, *linC*, *linX*, *pcpC*) and chloroalkane/chloroalkene (*adH*, *dehH*, *dhaA*, *adhP*) were detected. Also detected were degradative genes for toluene (*bbsG*, *bbsC*, *bbsD*, *tsaC1*), naphthalene (*adH*, *adhP*, *bnsG*), aminobenzoate (anthraniloyl-CoA monooxygenase, *ligC*, *lpdB*), ethylbenzene (*ped*, *etbD*), *dioxin* (*mhpF*, *bphD*), nitrotoluene (*nemA*) and several other aromatic hydrocarbons (Table 2). The benzoate and xylene degradation pathways, indicating the presence of the genes reported in this study and the reactions they catalysed in the pathways, are depicted in Fig. 4 and Fig. 5. In the FN4 metagenome, relatively few hydrocarbon degradation genes were detected. These include genes for 2-oxo-3-hexenedioate decarboxylase, 2-keto-4-pentenoate hydratase and 2-oxopent-4-enoate/cis-2-oxohex-4-enoate hydratase involved in dioxin, xylene and benzoate degradation. Others include putative genes for anthraniloyl-COA monooxygenase (aminobenzoate degradation), N-ethylmaleimide reductase (nitrotoluene degradation), bifunctional salicylyl-COA 5-hydroxylase/oxidoreductase (salicylate degradation) and 2-oxo-hepta-3-ene-1,7-dioic acid hydratase (*hpaH*) involved in 4-hydroxyphenylacetate and 2-oxopentenoate degradation.

**Table 2:** Putative hydrocarbon degradation genes detected in the FN1 metagenome and their taxonomic affiliation

Hydrocarbon degradation genes	Taxonomic affiliation
**Degradation of aromatic compounds**	
ko:K00001 E1.1.1.1; alcohol dehydrogenase [EC:1.1.1.1]	*Mesorhizobium denitrificans; Microbacterium lacticum*
ko:K01055 pcaD; 3-oxoadipate enol-lactonase [EC:3.1.1.24]	*Mesorhizobium albiziae*
ko:K04073 mhpF; acetaldehyde dehydrogenase [EC:1.2.1.10]	*Pelagibacterium* sp. SCN 64-44
ko:K05714 mhpC; 2-hydroxy-6-oxonona-2,4-dienedioate hydrolase [EC:3.7.1.14]	*Mesorhizobium albiziae*
ko:K05783 benD-xylL; dihydroxycyclohexadiene carboxylate dehydrogenase [EC:1.3.1.25 1.3.1.-]	*Rhizobiales* bacterium
ko:K05784 benC-xylZ; benzoate/toluate 1,2-dioxygenase reductase component [EC:1.18.1.-]	*Rhizobiales* bacterium
ko:K07535 badH; 2-hydroxycyclohexanecarboxyl-CoA dehydrogenase [EC:1.1.1.-]	*Candidatus Rokubacteria* bacterium; *Mesorhizobium denitrificans*; *Rhizobiales* bacterium
ko:K07538 had; 6-hydroxycyclohex-1-ene-1-carbonyl-CoA dehydrogenase [EC:1.1.1.368]	*Mesorhizobium denitrificans; Microbacterium lacticum*
ko:K07545 bbsG; (R)-benzylsuccinyl-CoA dehydrogenase [EC:1.3.8.3]	*Nocardioides* sp. dk3543
ko:K07547 bbsC; 2-[hydroxy(phenyl)methyl]-succinyl-CoA dehydrogenase BbsC subunit [EC:1.1.1.35]	*Rhizobiales* bacterium
ko:K07548 bbsD; 2-[hydroxy(phenyl)methyl]-succinyl-CoA dehydrogenase BbsD subunit [EC:1.1.1.35]	*Rhizobiales* bacterium
ko:K10216 dmpD; 2-hydroxymuconate-semialdehyde hydrolase [EC:3.7.1.9]	*Mesorhizobium albiziae*
ko:K10219 ligC; 2-hydroxy-4-carboxymuconate semialdehyde hemiacetal dehydrogenase [EC:1.1.1.312]	*Pelagibacterium* sp. SCN 64-44; *Devosia* sp. LC5; *Devosia elaeis*
ko:K10222 bphD; 2,6-dioxo-6-phenylhexa-3-enoate hydrolase [EC:3.7.1.8]	*Mesorhizobium albiziae*
ko:K10617 cymB; p-cumic alcohol dehydrogenase	*Rhizobiales* bacterium
ko:K10620 cmtB; 2,3-dihydroxy-2,3-dihydro-p-cumate dehydrogenase [EC:1.3.1.58]	*Rhizobiales* bacterium
ko:K13953 adhP; alcohol dehydrogenase, propanol-preferring [EC:1.1.1.1]	*Mesorhizobium denitrificans; Microbacterium lacticum*
ko:K14727 pcaL; 3-oxoadipate enol-lactonase / 4-carboxymuconolactone decarboxylase [EC:3.1.1.24 4.1.1.44]	*Mesorhizobium albiziae*
ko:K14746 ped; (S)-1-phenylethanol dehydrogenase [EC:1.1.1.311]	*Rhizobiales* bacterium
ko:K15571 bnsG; naphthyl-2-methylsuccinyl-CoA dehydrogenase [EC:1.3.99.-]	*Nocardioides* sp. dk3543
ko:K17754 cpnA; cyclopentanol dehydrogenase [EC:1.1.1.163]	*Rhizobiales* bacterium
ko:K18067 pht4; phthalate 4,5-cis-dihydrodiol dehydrogenase [EC:1.3.1.64]	*Pelagibacterium* sp. SCN 64-44; *Devosia* sp. LC5; *Devosia elaeis*
ko:K18092 etbD; 2-hydroxy-6-oxo-octa-2,4-dienoate hydrolase [EC:3.7.1.-]	*Mesorhizobium albiziae*
ko:K19960 chnA; cyclohexanol dehydrogenase [EC:1.1.1.245]	*Rhizobiales* bacterium
**Benzoate degradation**	
ko:K01055 pcaD; 3-oxoadipate enol-lactonase [EC:3.1.1.24]	*Mesorhizobium albiziae*
ko:K04073 mhpF; acetaldehyde dehydrogenase [EC:1.2.1.10]	*Pelagibacterium* sp. SCN 64-44
ko:K04117 aliB; cyclohexanecarboxyl-CoA dehydrogenase [EC:1.3.99.-]	*Nocardioides* sp.
ko:K04118 E1.3.1.62; pimeloyl-CoA dehydrogenase [EC:1.3.1.62]	*Nocardioides* sp.
ko:K05783 benD-xylL; dihydroxycyclohexadiene carboxylate dehydrogenase [EC:1.3.1.25 1.3.1.-]	*Rhizobiales* bacterium
ko:K05784 benC-xylZ; benzoate/toluate 1,2-dioxygenase reductase component [EC:1.18.1.-]	*Rhizobiales* bacterium
ko:K07535 badH; 2-hydroxycyclohexanecarboxyl-CoA dehydrogenase [EC:1.1.1.-]	*Candidatus Rokubacteria* bacterium; *Mesorhizobium denitrificans*; *Rhizobiales* bacterium
ko:K07538 had; 6-hydroxycyclohex-1-ene-1-carbonyl-CoA dehydrogenase [EC:1.1.1.368]	*Mesorhizobium denitrificans; Microbacterium lacticum*
ko:K10216 dmpD; 2-hydroxymuconate-semialdehyde hydrolase [EC:3.7.1.9]	*Mesorhizobium albiziae*
ko:K10219 ligC; 2-hydroxy-4-carboxymuconate semialdehyde hemiacetal dehydrogenase [EC:1.1.1.312]	*Pelagibacterium* sp. SCN 64-44; *Devosia* sp. LC5; *Devosia elaeis*
ko:K14334 CMLE; carboxy-cis,cis-muconate cyclase [EC:5.5.1.5]	*Mesorhizobium* sp.; *Mesorhizobium prunaredense; Mesorhizobium* sp. M2A.F.Ca.ET.042.01.1.1;
ko:K14727 pcaL; 3-oxoadipate enol-lactonase / 4-carboxymuconolactone decarboxylase [EC:3.1.1.24 4.1.1.44]	*Mesorhizobium albiziae*
ko:K16173 acd; glutaryl-CoA dehydrogenase (non-decarboxylating) [EC:1.3.99.32]	*Nocardioides* sp. dk3543
ko:K19066 E1.3.8.10; cyclohex-1-ene-1-carbonyl-CoA dehydrogenase [EC:1.3.8.10]	*Nocardioides* sp. dk3543
ko:K19067 E1.3.8.11; cyclohexane-1-carbonyl-CoA dehydrogenase [EC:1.3.8.11]	*Nocardioides* sp. dk3543
**Xylene degradation**	
ko:K04073 mhpF; acetaldehyde dehydrogenase [EC:1.2.1.10]	*Pelagibacterium* sp. SCN 64-44
ko:K05783 benD-xylL; dihydroxycyclohexadiene carboxylate dehydrogenase [EC:1.3.1.25 1.3.1.-]	*Rhizobiales* bacterium
ko:K05784 benC-xylZ; benzoate/toluate 1,2-dioxygenase reductase component [EC:1.18.1.-]	*Rhizobiales* bacterium
ko:K10216 dmpD; 2-hydroxymuconate-semialdehyde hydrolase [EC:3.7.1.9]	*Mesorhizobium albiziae*
ko:K10617 cymB; p-cumic alcohol dehydrogenase	*Rhizobiales* bacterium
ko:K10620 cmtB; 2,3-dihydroxy-2,3-dihydro-p-cumate dehydrogenase [EC:1.3.1.58]	*Rhizobiales* bacterium
**Chlorocyclohexane and chlorobenzene degradation**	
ko:K01561 dehH; haloacetate dehalogenase [EC:3.8.1.3]	*Mesorhizobium albiziae*
ko:K01563 dhaA; haloalkane dehalogenase [EC:3.8.1.5]	*Mesorhizobium albiziae*
ko:K15237 linC; 2,5-dichloro-2,5-cyclohexadiene-1,4-diol dehydrogenase 1 [EC:1.3.1.-]	*Mesorhizobium denitrificans; Rhizobiales* bacterium
ko:K15238 linX; 2,5-dichloro-2,5-cyclohexadiene-1,4-diol dehydrogenase 2 [EC:1.3.1.-]	*Rhizobiales* bacterium
ko:K15241 pcpC; tetrachlorohydroquinone reductive dehalogenase [EC:1.21.4.5]	*Devosia* sp. I507
**Chloroalkane and chloroalkene degradation**	
ko:K00001 E1.1.1.1; alcohol dehydrogenase [EC:1.1.1.1]	*Mesorhizobium denitrificans; Microbacterium lacticum*
ko:K01561 dehH; haloacetate dehalogenase [EC:3.8.1.3]	*Mesorhizobium albiziae*
ko:K01563 dhaA; haloalkane dehalogenase [EC:3.8.1.5]	*Mesorhizobium albiziae*
ko:K13953 adhP; alcohol dehydrogenase, propanol-preferring [EC:1.1.1.1]	*Mesorhizobium denitrificans; Microbacterium lacticum*
**Toluene degradation**	
ko:K07545 bbsG; (R)-benzylsuccinyl-CoA dehydrogenase [EC:1.3.8.3]	*Nocardioides* sp. dk3543
ko:K07547 bbsC; 2-[hydroxy(phenyl)methyl]-succinyl-CoA dehydrogenase BbsC subunit [EC:1.1.1.35]	*Rhizobiales* bacterium
ko:K07548 bbsD; 2-[hydroxy(phenyl)methyl]-succinyl-CoA dehydrogenase BbsD subunit [EC:1.1.1.35]	*Rhizobiales* bacterium
ko:K19630 tsaC1; 4-formylbenzenesulfonate dehydrogenase [EC:1.2.1.62]	*Rhizobiales* bacterium
**Naphthalene degradation**	
ko:K00001 E1.1.1.1; alcohol dehydrogenase [EC:1.1.1.1]	*Mesorhizobium denitrificans; Microbacterium lacticum*
ko:K13953 adhP; alcohol dehydrogenase, propanol-preferring [EC:1.1.1.1]	*Mesorhizobium denitrificans; Microbacterium lacticum*
ko:K15571 bnsG; naphthyl-2-methylsuccinyl-CoA dehydrogenase [EC:1.3.99.-]	*Nocardioides* sp. dk3543
**Aminobenzoate degradation**	
ko:K09461 E1.14.13.40; anthraniloyl-CoA monooxygenase [EC:1.14.13.40]	*Azospirillum* sp. TSA6c; *Azospirillum doebereinerae*
ko:K10219 ligC; 2-hydroxy-4-carboxymuconate semialdehyde hemiacetal dehydrogenase [EC:1.1.1.312]	*Pelagibacterium* sp. SCN 64-44; *Devosia* sp. LC5; *Devosia elaeis*
ko:K22959 lpdB; gallate decarboxylase subunit B	*Pseudaminobacter* sp. CB3; *Hyphomicrobiaceae* bacterium TMED74
**Ethylbenzene degradation**	
ko:K14746 ped; (S)-1-phenylethanol dehydrogenase [EC:1.1.1.311]	*Rhizobiales* bacterium
ko:K18092 etbD; 2-hydroxy-6-oxo-octa-2,4-dienoate hydrolase [EC:3.7.1.-]	*Mesorhizobium albiziae*
**Nitrotoluene degradation**	
ko:K10680 nemA; N-ethylmaleimide reductase [EC:1.-.-.-]	*Azospirillum* sp. TSA6c; *Azospirillum doebereinerae*

Putative genes for uptake, transport, efflux and regulation of various inorganic nutrients and heavy metals were detected in FN1 metagenome (Table 3). These include genes for transport, uptake and regulation of phosphate/phosphonate (*pstB*, *pstS*, *phnC*, *phoB*, *ompR*), nitrogen (*urtD*, *urtE*, *nrtC, nrtD*, *glnC*, *ntrY*, *ntrX*), sufate/thiosulfate (*cysA*, *cysP*, *ssuB*, *sbp*) and several others. Putative genes for uptake, transport, efflux and regulation of heavy metals such as cobalt/nickel (*cbiO, nikD*, *nikE*, *nrsR*), iron (*afuC*, *afuA*, *fhuC*, *fecE*, *fepC*, *fepA*), molybdate/tungstate (*modA*, *modB*, *modC*, *modF*, *tupC*, *wtpC*), manganese/zinc/iron (*znuC*, *psaB*, *mntB*, *mntA*, *sitB*, *troB*, *manR*) and copper (*nosF*, *cusR*) were also detected (Table 3). In the FN4 metagenome, putative genes for transport, uptake and regulation of inorganic nutrients such as phosphate/phosphonate (*phoB*, *phnC*, *ompR*) and nitrogen (*narL*, *narP*, *ntrX*, *glnG*) were detected. For heavy metals, putative genes for transport, efflux and regulation such as *manR* (manganese), *cusR* (copper), *afuC* (iron) and *modC* (molybdate) were also detected.

**Table 3:** Putative genes for ABC transporters and their two-component systems detected in the FN1 metagenome and their taxonomic affiliation

Putative genes	Taxonomic affiliation
**ABC transporters**	
ko:K02006 cbiO; cobalt/nickel transport system ATP-binding protein	*Listeria monocytogenes; Zooshikella ganghwensis*; *Azospirillum* sp. (strain B510); *Raoultella* sp. BIGb0138; *Chelatococcus* sp. CO-6; *Alteromonadaceae* bacterium 2052S.S.stab0a.01; *Jiella endophytica*
ko:K02010 afuC; iron(III) transport system ATP-binding protein [EC:7.2.2.7]	*Azospirillum* sp. (strain B510); *Raoultella* sp. BIGb0138; *Chelatococcus* sp. CO-6; *Alteromonadaceae* bacterium 2052S.S.stab0a.01
ko:K02012 afuA; iron(III) transport system substrate-binding protein	*Mesorhizobium denitrificans; Mesorhizobium* sp. YM1C-6-2
ko:K02017 modC; molybdate transport system ATP-binding protein [EC:7.3.2.5]	*Zooshikella ganghwensis; Azospirillum* sp. (strain B510); *Chelatococcus* sp. CO-6; *Alteromonadaceae* bacterium 2052S.S.stab0a.01
ko:K02018 modB; molybdate transport system permease protein	*Listeria monocytogenes*; *Zooshikella ganghwensis*; *Alteromonadaceae* bacterium 2052S.S.stab0a.01
ko:K02020 modA; molybdate transport system substrate-binding protein	*Mesorhizobium denitrificans; Mesorhizobium* sp. YM1C-6-2
ko:K02036 pstB; phosphate transport system ATP-binding protein [EC:7.3.2.1]	*Zooshikella ganghwensis*; *Azospirillum* sp. (strain B510); *Chelatococcus* sp. CO-6; *Alteromonadaceae* bacterium 2052S.S.stab0a.01; *Jiella endophytica*
ko:K02040 pstS; phosphate transport system substrate-binding protein	Mesorhizobium denitrificans
ko:K02041 phnC; phosphonate transport system ATP-binding protein [EC:7.3.2.2]	*Zooshikella ganghwensis*; *Azospirillum* sp. (strain B510); *Raoultella* sp. BIGb0138; *Chelatococcus* sp. CO-6; *Alteromonadaceae* bacterium 2052S.S.stab0a.01; *Jiella endophytica*
ko:K02045 cysA; sulfate/thiosulfate transport system ATP-binding protein [EC:7.3.2.3]	*Zooshikella ganghwensis*; *Azospirillum* sp. (strain B510); *Chelatococcus* sp. CO-6; *Alteromonadaceae* bacterium 2052S.S.stab0a.01
ko:K02048 cysP; sulfate/thiosulfate transport system substrate-binding protein	*Mesorhizobium denitrificans; Mesorhizobium* sp. YM1C-6-2
ko:K05776 modF; molybdate transport system ATP-binding protein	*Listeria monocytogenes; Zooshikella ganghwensis*; *Chelatococcus* sp. CO-6; *Alteromonadaceae* bacterium 2052S.S.stab0a.01
ko:K06857 tupC; tungstate transport system ATP-binding protein [EC:7.3.2.6]	*Listeria monocytogenes; Zooshikella ganghwensis*; *Azospirillum* sp. (strain B510); *Chelatococcus* sp. CO-6; *Alteromonadaceae* bacterium 2052S.S.stab0a.01; *Jiella endophytica*
ko:K09817 znuC; zinc transport system ATP-binding protein [EC:7.2.2.-]	*Listeria monocytogenes; Zooshikella ganghwensis*; *Azospirillum* sp. (strain B510); *Raoultella* sp. BIGb0138; *Chelatococcus* sp. CO-6; *Alteromonadaceae* bacterium 2052S.S.stab0a.01; *Jiella endophytica*
ko:K10824 nikE; nickel transport system ATP-binding protein [EC:7.2.2.11]	*Zooshikella ganghwensis*; *Azospirillum* sp. (strain B510); *Chelatococcus* sp. CO-6; *Alteromonadaceae* bacterium 2052S.S.stab0a.01
ko:K10829 fhuC; ferric hydroxamate transport system ATP-binding protein [EC:7.2.2.16]	*Zooshikella ganghwensis*; *Azospirillum* sp. (strain B510); *Chelatococcus* sp. CO-6; *Alteromonadaceae* bacterium 2052S.S.stab0a.01; *Jiella endophytica*
ko:K10830 psaB; manganese/zinc transport system ATP-binding protein [EC:7.2.2.5]	*Zooshikella ganghwensis*; *Azospirillum* sp. (strain B510); *Chelatococcus* sp. CO-6; *Alteromonadaceae* bacterium 2052S.S.stab0a.01
ko:K11602 mntB; manganese transport system permease protein	*Microbacterium* sp. cf046
ko:K11603 mntA; manganese transport system ATP-binding protein [EC:7.2.2.5]	*Zooshikella ganghwensis; Azospirillum* sp. (strain B510); *Chelatococcus* sp. CO-6; *Alteromonadaceae* bacterium 2052S.S.stab0a.01
ko:K11607 sitB; manganese/iron transport system ATP-binding protein	*Zooshikella ganghwensis*; *Azospirillum* sp. (strain B510); *Raoultella* sp. BIGb0138; *Chelatococcus* sp. CO-6; *Alteromonadaceae* bacterium 2052S.S.stab0a.01
ko:K11710 troB; manganese/zinc/iron transport system ATP- binding protein [EC:7.2.2.5]	*Zooshikella ganghwensis*; *Azospirillum* sp. (strain B510); *Chelatococcus* sp. CO-6; *Alteromonadaceae* bacterium 2052S.S.stab0a.01
ko:K11962 urtD; urea transport system ATP-binding protein	*Zooshikella ganghwensis*; *Chelatococcus* sp. CO-6; *Alteromonadaceae* bacterium 2052S.S.stab0a.01
ko:K11963 urtE; urea transport system ATP-binding protein	*Zooshikella ganghwensis*; *Chelatococcus* sp. CO-6; *Alteromonadaceae* bacterium 2052S.S.stab0a.01; *Jiella endophytica*
ko:K15497 wtpC; molybdate/tungstate transport system ATP-binding protein [EC:7.3.2.5 7.3.2.6]	*Listeria monocytogenes; Zooshikella ganghwensis*; *Azospirillum* sp. (strain B510); *Chelatococcus* sp. CO-6; *Alteromonadaceae* bacterium 2052S.S.stab0a.01; *Jiella endophytica*
ko:K15555 ssuB; sulfonate transport system ATP-binding protein [EC:3.6.3.-]	*Zooshikella ganghwensis*; *Azospirillum* sp. (strain B510); *Chelatococcus* sp. CO-6; *Alteromonadaceae* bacterium 2052S.S.stab0a.01;
ko:K15558 ophH; phthalate transport system ATP-binding protein	*Chelatococcus* sp. CO-6
ko:K15578 nrtC; nitrate/nitrite transport system ATP-binding protein [EC:3.6.3.-]	*Azospirillum* sp. (strain B510); *Raoultella* sp. BIGb0138; *Chelatococcus* sp. CO-6
ko:K15579 nrtD; nitrate/nitrite transport system ATP-binding protein	*Azospirillum* sp. (strain B510); *Chelatococcus* sp. CO-6; *Alteromonadaceae* bacterium 2052S.S.stab0a.01
ko:K15587 nikD; nickel transport system ATP-binding protein [EC:7.2.2.11]	*Listeria monocytogenes; Zooshikella ganghwensis*; *Azospirillum* sp. (strain B510); *Chelatococcus* sp. CO-6; *Alteromonadaceae* bacterium 2052S.S.stab0a.01
ko:K18895 iroC; ATP-binding cassette, subfamily B, salmochelin/enterobactin exporter	*Azospirillum* sp. (strain B510); *Chelatococcus* sp. CO-6
ko:K19340 nosF; Cu-processing system ATP-binding protein	*Zooshikella ganghwensis*; *Azospirillum* sp. (strain B510); *Chelatococcus* sp. CO-6; *Alteromonadaceae* bacterium 2052S.S.stab0a.01
ko:K23163 sbp; sulfate/thiosulfate transport system substrate-binding protein	*Mesorhizobium denitrificans; Mesorhizobium* sp. YM1C-6-2
ko:K23184 fecE; ferric citrate transport system ATP-binding protein [EC:7.2.2.18]	*Zooshikella ganghwensis*; *Chelatococcus* sp. CO-6; *Alteromonadaceae* bacterium 2052S.S.stab0a.01; *Jiella endophytica*
ko:K23188 fepC; ferric enterobactin transport system ATP-binding protein [EC:7.2.2.17]	*Zooshikella ganghwensis*; *Chelatococcus* sp. CO-6; *Alteromonadaceae* bacterium 2052S.S.stab0a.01
**Two-component systems**	
ko:K02040 pstS; phosphate transport system substrate-binding protein	*Mesorhizobium denitrificans*
ko:K07657 phoB; two-component system, OmpR family, phosphate regulon response regulator PhoB	*Burkholderiales* bacterium
ko:K07659 ompR; two-component system, OmpR family, phosphate regulon response regulator OmpR	*Burkholderiales* bacterium
ko:K07665 cusR; two-component system, OmpR family, copper resistance phosphate regulon response regulator CusR	*Burkholderiales* bacterium
ko:K07667 kdpE; two-component system, OmpR family, KDP operon response regulator KdpE	*Nocardioides* sp.; *Burkholderiales* bacterium
ko:K07708 glnL; two-component system, NtrC family, nitrogen regulation sensor histidine kinase GlnL [EC:2.7.13.3]	*Nocardioides* sp.; *Alcaligenes xylosoxydans xylosoxydans*; *Streptomyces* sp. SLBN-118
ko:K11330 nrsR; two-component system, OmpR family, Ni(II)-responsive and/or redox-responsive regulator NrsR	*Burkholderiales* bacterium
ko:K11521 manR; two-component system, OmpR family, manganese sensing response regulator	*Burkholderiales* bacterium
ko:K11602 mntB; manganese transport system permease protein	*Microbacterium* sp. cf046
ko:K11603 mntA; manganese transport system ATP-binding protein [EC:7.2.2.5]	*Zooshikella ganghwensis*; *Azospirillum* sp. (strain B510); *Chelatococcus* sp. CO-6; *Alteromonadaceae* bacterium 2052S.S.stab0a.01
ko:K13598 ntrY; two-component system, NtrC family, nitrogen regulation sensor histidine kinase NtrY [EC:2.7.13.3]	*Nocardioides* sp.; *Streptomyces* sp. SLBN-118
ko:K13599 ntrX; two-component system, NtrC family, nitrogen regulation response regulator NtrX	*Burkholderiales* bacterium; *Chelatococcus* sp. CO-6
ko:K19611 fepA; ferric enterobactin receptor	*Gemmatimonadales* bacterium

Worth mentioning is the detection of genes responsible for biosynthesis of biosurfactants produced by members of the microbial community in FN1 metagenome. These include rhamnosyltransferase subunit B (*rhlB*), a member of *rhlAB* gene responsible for rhamnolipid biosynthesis, and phosphatidyl-N-methylethanolamine N-methyltransferase responsible for biosynthesis of a phospholipid biosurfactant, phosphatidylethanolamine. It is instructive to note that these genes were not detected in the FN4 metagenome. Putative genes responsible for bacterial chemotaxis were also detected from the two metagenomes. Putative genes for bacterial chemotaxis proteins *cheR*, *cheB*, *cheBR*, *cheY*, *aer*, *motB* and *rbsB* were detected in FN1 while *cheB*, *cheY*, *cheV* and *cheBR* were detected in the FN4 metagenome.

## Discussion

In order to be able to correctly assess the impact of a pollutant on an environmental compartment and proffer an appropriate strategy for reclamation, it is necessary to embark on a holistic and optimizable characterization of such site. Monitoring of trends in pollutant depletion and shifts in microbial community structures and functions relying especially on standard analytical chemistry methods and molecular approaches that enable access to both culturable and non-culturable phylotypes has gained currency as the best practice in this regard.

Analysis of the animal charcoal-polluted soil revealed the presence of heavy metals, low inorganic nutrients and an acidic pH. The acidic pH of the polluted system is a clear indication of active catalytic activities of the autochthonous community on diverse hydrocarbons and organic materials present in the soil, which yield acidic end products [25]. However, in such acidic systems, there is a trade-off in diversity of phylotypes directly involved in the process as biodegradation of hydrocarbons by microorganisms has been shown to proceed relatively fast under conditions close to neutrality [3, 26].

In an actively metabolizing microbial community, inorganic nutrients are always a limiting factor as they are required by members of the community for vital cellular functions and metabolic activities. This possibly explains the low concentrations of nitrogen and phosphorus in the polluted system. Functional annotation of the two metagenomes, FN1 and FN4, further revealed the limiting nature of these nutrients. For instance, the *pstB* and *pstS* are part of periplasmic transport system which are respectively ATP-binding and ATP-hydrolysis sites [27]. The *phnC* which was annotated for several species in the FN1 metagenome is encoded by the *phn* operon which is a member of the Pho regulon normally induced under phosphate starvation [28]. This suggests that these organisms were able to circumvent the shortage of inorganic phosphate in the polluted soil by activating the genes for acquisition and metabolism of less readily available organic sources requiring the activity of phosphate-carbon lyase. This is further buttressed by detection of two-component system genes *phoB ompR* and *cusR* which are part of the transduction systems that enable sense, respond and adapt to changes in their environment. While these genes are also annotated in FN4, it is only affiliated to *Klebsiella* sp., thus suggesting that phosphorus starvation is not prevalent in FN4, possibly due to addition of CFMM.

Similarly, while the two metagenomes, FN1 and FN4, were annotated for genes involved in inorganic nitrogen metabolism such as nitrate/nitrite transporter systems, the genes for urea transport were only annotated in FN1. This is interesting as the *urtD* and *urtE* genes are part of the genes normally activated in nitrogen limited environment for utilization of urea as nitrogen source and have been reported to be activated under such condition in some hydrocarbon degraders [29]. The absence of the *urtD* and *urtE* genes in FN4 could only be attributed to the addition of CFMM, which provide the needed inorganic nitrogen, thus making it impracticable due to energy costs to use urea as nitrogen source. This possibly results in shutting down the metabolic pathway for urea transport and metabolism via feedback inhibition.

The detection of the putative genes for regulation, transport and efflux of heavy metals namely those for cobalt/nickel, molybdate/tungstate and manganese/zinc/iron underscores the importance of these metals both as stressors and as nutrient requirements of the microbial community. Heavy metals are common co-contaminants of hydrocarbon-polluted sites arising as components of crude oil or acquired in the process of use, transport and disposal [30–32]. In excess of tolerance levels, heavy metals impose several inimical influences on microbial cells including cell membrane disruption, DNA damage, protein denaturation and inhibition of transcription, translation, enzyme activity and cell division. Several resistance mechanisms adopted to surmount this include internal and external sequestration, biosurfactant production, volatilization, precipitation and efflux pump systems [33–35]. It is equally noteworthy that most of the genes putatively identified in FN1 spanned various genera in the microcosm suggesting that they must have been acquired by and spread through the community horizontal gene transfer via mobile genetic elements [15, 36, 37].

It is noteworthy that the level of hydrocarbon pollution observed in the soil sample is more than the 500 mg/kg limit established by regulatory bodies [4], although far less than the value of 2057.55 mg/kg we earlier reported for a similar site in Lagos, Nigeria [3]. In both FN1 and FN4 microcosms, there were considerable decreases in total hydrocarbon content during the 42-day treatability period, indicating that there were autochthonous populations with hydrocarbon metabolism capability in the system. However, the fact that over 70% reduction in aliphatic and aromatic hydrocarbons was observed in FN1 where there was only addition of water and tilling highlights the important role of water activity as a limiting factor in the biodegradation of hydrocarbon pollutants in soil, especially in environments such as arid soil where moisture content is low or where water activity is low [38]. This trend has also been observed in our previous study [39]. Furthermore, it is not always the case that nutrient addition dramatically increases rate of degradation of hydrocarbons in soil. In some cases, nutrient addition had been reported to lead to insignificant or even negative outcomes [40, 41]. However, the initial more rapid rate of removal of aliphatic and aromatic hydrocarbons in the FN4 microcosm in the first 21 days and subsequent overall higher total reduction is evidence of the stimulatory effect of the added CFMM.

The early disappearance of the lower-molecular-weight hydrocarbons, ethane, propane, cyclopropane, butane, methylpropane, pentane, methylbutane and tricosane in both microcosm and complete disappearance of hexane and octane in FN4 within the first 21 days are not surprising. These are low molecular-weight aliphatic fractions which are known to readily lend themselves to microbial degradation than the aromatic fractions [42, 43]. However, it is not unlikely that some of the disappearance might be abiotic resulting from volatilization. The complete disappearance of all other aliphatics with a few exceptions, and complete disappearance of naphthalene, fluoranthene, benzo(a)pyrene and indeno(123-cd)pyrene in the FN4 after 42 days are noteworthy. This coupled with the fact that higher percentage removal was recorded for all other aromatic fractions including recalcitrant pyrene and chrysene in FN4 further demonstrated the role of nutrient amendment. Indeed, whereas the ability of bacteria in the environment to degrade the low-molecular-weight PAHs is widespread, the same is not the case with fused four- and five-ring PAHs [44].

Metagenomic analysis showed remarkable difference in the diversity of the communities at the phyla level in FN1 and FN4, with the former having 14 phyla represented and the CFMM-treated FN4 having representation in 8 phyla. The fact that the three most prominent phyla in FN1 are *Proteobacteria* (56.12%), *Actinobacteria* (23.79%) and *Firmicutes* (11.20%) is not surprising as previous reports had shown *Proteobacteria* and *Actinobacteria* to be the phyla more readily adapted to hydrocarbon-polluted matrices, basically on account of their broad substrate specificity for diverse classes of hydrocarbons and ability to survive in harsh, oligotrophic environments [45–51].

It is however interesting that many of the phyla which were represented in the FN1 soil completely disappeared in the FN4, leaving mainly the three mentioned above, with the *Firmicutes* accounting for more than 90%. Though the expectation was that nutrient addition would stimulate the dominant hydrocarbon degraders to blossom and express their functionality, it has been shown that bacteria richness and diversity in soil could be decreased when the soil is enriched with nitrogen and phosphorus [52]. The dominant role of *Firmicutes* may not be unconnected with other hidden environmental factors that may limit the ability of the *Proteobacteria* and *Actinobacteria* to take advantage of the new nutrient regime. It is equally noteworthy that many *Actinobacteria* generally are oligotrophs and slow growers which can easily be overtaken by copiotrophs and are favoured in an environment with pH 6 and 9 [53, 54]. Several authors have established that physicochemical parameters play a cardinal role in determining the community composition of hydrocarbon impacted systems and even a slight alteration in certain parameters could significantly skew the distribution in favour of a particular group [38, 55].

At the class level, the *Alphaproteobacteria* dominated the FN1 microcosm followed by the *Actinobacteria* and *Gammaproteobacteria*. The two *Proteobacteria* classes are a group imbued with a variety of metabolic strategies, including nitrogen fixation, ammonia oxidation, chemoautotrophy, methylotrophy and temperature adaptation amongst others [56, 57]. It is therefore not unlikely that rather than nutrients such as nitrogen and phosphorus, water alone constituted the most important limiting factor. The preponderance of *Rhizobiales* (26.30%) at the order level and the *Xanthobacteriaceae* (10.94%) and *Rhizobiaceae* (7.9 %) at the family level seems to further buttress this, as the prominence of these groups in the ecosystem where available nutrients were depleted as a result of hydrocarbon contamination has been reported [58]. The predominant phenotypes at the genus level, namely, *Xanthobacter* (9.73%), *Rhizobium* (7.49%) and *Corynebacterium* (7.35%), have strains which have been well reported in the biodegradation of both aliphatic and aromatic fractions in hydrocarbon-polluted soils [59–61].

The two most represented genera in the CFMM-amended FN4 microcosm were *Anoxybacillus* (64.58%) and *Bacillus* (21.47%) both of which are members of the family *Bacillaceae* in the order *Bacillales*. They are both endospore-forming Gram-positive rods. Predominance of the *Anoxybacillus* in FN4 is of interest because species of this unique genus have been isolated from diverse extreme environments including anoxic, geothermal springs and heavy metal-rich systems [62–65]. Although mostly alkali-tolerant thermophiles, they have been found to be physiologically diverse with respect to optimal temperature of growth and pH, with some being aerobic and others anaerobic [63]. While the genus *Anoxybacillus* are not recognized as a prominent player in the degradation of hydrocarbons, Al-Jailawi et al. [66] reported a thermophilic *Anoxybacillus rupeinsis* strain Ir3 isolated from a hydrocarbon-polluted site in Iran with metabolic propensity for aromatic compounds including carbazole, ρ-nitrophenol, nitrobenzene and naphthalene. The upscaling of *Anoxybacillus* as the predominant genus in the CFMM-treated animal charcoal-polluted soil FN4 may not be unconnected to the fact that the soil represented a localized extreme environment in which most other potential hydrocarbon degraders were not favoured. Furthermore, it is also not unlikely that *Anoxybacillus* strains were able to take advantage of the nutrient addition than their potential rivals.

*Bacillus* is the largest and best studied genus in terms of the number of isolates, studies conducted and published reports among the *Bacillaceae* [67, 68]. It is a long-standing genus in terms of hydrocarbon degradation capability, and reports abound in the literature of its metabolic capabilities on saturated, aromatic and heteroaromatic hydrocarbon fractions [69]. Therefore, detection of *Bacillus* as the second most represented genus in the FN4 microcosm is not entirely surprising. *Solibacillus*, which is the third (2.39%) most preponderant genus in the FN4 soil, is a small group in the family *Planococcaceae*. Only three rarely isolated species are known, namely *S. silvestris*, *S. isorensis* [70, 71] and *S. kalamii* [72]. Members of the genus have been isolated from cryotube used for sampling the upper atmosphere, high-efficiency particulate arrestance (HEPA) filter, and identified as key colonizers of biofilter in a waste gas treatment plant [72, 73]. Evidently, the *Solibacillus* appear to be organisms adapted to high-stressed environments [74]. It is therefore not surprising that they are making an impressive appearance in such a unique and little understood ecosystem as the soil of cottage industry producing ponmo. Thus, the biostimulation with CFMM dramatically altered the community structure in favour of unique, rarely isolated and physiologically versatile strains, which deserve to be further explored by isolating and properly characterizing them.

Hydrocarbon-polluted sites are usually co-contaminated with other xenobiotic compounds and heavy metals. This usually triggers avalanche of responses from the autochthonous microbial community to counteract the negative impacts of the environmental stressors. The metabolic functions highlighted in the genes identified in FN1 metagenome not only reaffirm the cocktail of pollutants with which the animal charcoal site is laced alongside the hydrocarbons but is also a reflection of the metabolism of these pollutants by the resident flora [75]. It is equally not surprising that the groups of organisms belonging to the order *Rhizobales* (particularly *Rhizobium* and *Mesorhizobium*) and *Actinobacteria* which were the predominant taxa were also the ones annotated for the metabolic functions.

The rapid disappearance of short-chained aliphatics in FN1 microcosm may not be unconnected to the activity of alcohol dehydrogenase, which was detected in this study. The alcohol dehydrogenase catalysed step is a very important one in the metabolic processing of aliphatic hydrocarbons and side chain reactions of aromatics and their derivatives. The enzyme has a broad substrate specificity on various classes of aliphatic, alicyclic and aromatic alcohols and hydroxyls [76–79].

The initial step in the aerobic degradation of aromatic compound usually involves the dihydroxylation of one of the polynuclear aromatic rings by incorporation of two atoms of oxygen into the aromatic ring. This process activates the molecule compromising its integrity as it prepares it for cleavage. Ring hydroxylation is catalysed by a multi-component dioxygenase which consists of a reductive, a ferredoxin and an iron sulfur protein, while ring cleavage is generally catalysed by an iron containing *meta* cleavage enzyme. The carbon skeleton produced by the ring cleavage reaction is then dismantled, before cleavage of the second aromatic ring [80]. The subsequent steps involve a number of enzymes which catalyse a range of reactions including hydroxylation and cleavage and eventual processing through either an *ortho* or a *meta* cleavage type of pathway, leading to central intermediates such as protocatechuate and catechol, which are further converted to tricarboxylic acid cycle intermediate [81, 82].

In this study, many of the annotated genes encoding enzymes (dehydrogenases, dioxygenase-reductases, hydrolases, decarboxylases, lactonases) that channelled the metabolic intermediates resulting from degradation of aromatics, particularly benzoates, xylene, toluene, naphthalene and ethylbenzene into the tricarboxylic acid were affiliated to the *Rhizobales* (*Mesorhizobium*) and *Nocardioides*. Thus, it appears that while other players no doubt contributed to the degradation of these compounds, the aforementioned genera must have recruited over time most of the genetic capability for utilization of these aromatic compounds. Of interest are the genes annotated for benzoate degradation in *Nocardioides*, namely cyclohexanecarboxyl-CoA dehydrogenase, pimeloyl-CoA dehydrogenase, glutaryl-CoA dehydrogenase (non-decarboxylating), cyclohex-1-ene-1-carbonyl-CoA dehydrogenase, cyclohexane-1-carbonyl-CoA dehydrogenase and those annotated for *Candidatus Rokubacteria*, *Microbacterium* and *Rhizobales* namely dihydroxycyclohexadiene carboxylate dehydrogenase (*benD-xylL*), 2-hydroxycyclohexanecarboxyl-CoA (*badH*) and 6-hydroxycyclohex-1-ene-1-carbonyl-CoA dehydrogenase (*had*). These are enzymes involved in the syntrophic production of cyclohexane carboxylate and acetate in the presence of benzoate [83, 84], which suggests that syntrophism, in which *Actinobacteria* such as *Nocardioides*, *Microbacterium* and *Candidatus Rokubacteria* are key symbionts, is an important interaction that facilitate pollutant removal in the FN1 animal charcoal microcosm.

The annotation of the genes encoding haloacetate dehalogenase (*DehH*), haloalkane dehalogenase (*dhaA*), 2,5-dichloro-2,5-cyclohexadiene-1,4-diol dehydrogenase 1 (*linC*) and 2,5-dichloro-2,5-cyclohexadiene-1,4-diol dehydrogenase 2 (*linX*) in relation to chlorocyclohexane, chlorobenzene, chloroalkane and chloroalkene metabolism is not surprising as the dehalogenation steps are usually important in the detoxification and ultimate mineralization of halogenated hydrocarbons. This group of enzymes catalyses the cleavage of the carbon-halogen bond of organohalogen compounds and has potential applications in the chemical industry and bioremediation. Hydrolytic dehalogenation is commonly performed by haloalkane dehalogenase, 2-haloacid dehalogenase and 4-chlorobenzoyl-CoA dehalogenase, while reductive respiratory dehalogenation is carried out by organisms which derive energy from the dehalogenation process [85, 86]. Haloalkane dehalogenases convert haloalkanes to their corresponding alkanes, halides and protons [87, 88]. These genes, like most genes associated with the degradation of xenobiotics, are acquired by horizontal transfer and localized on mobile genetic elements like plasmids over time. Presence of these genes in the metagenome suggests chronic contamination of the site with halogenated compounds.

Genes encoding some steps in the degradation of aminobenzoate were also annotated in the FN1 metagenome including anthraniloyl-CoA monooxygenase, 2-hydroxy-4-carboxymuconate semialdehyde hemiacetal dehydrogenase (*ligC*) and gallate decarboxylase subunit B (*lpdB*). 2-Aminobenzoate is a derivative of tryptophan and other indole-containing compounds. It is one of the intermediates in the degradation of *orho* nitrobenzoate and carbazole. One way apart from direct processing to catechol, by which it is aerobically metabolized, is the activation to 2-aminobenzoyl-CoA by anthranilate CoA ligase. The oxidoreductase enzyme anthraniloyl-CoA monooxygenase oxidizes this into 2-amino-5-oxo-cyclohex-1-enecarboxy-CoA [89]. Similarly, the annotation of *nemA*-N-ethylmaleimide reductase affiliated with *Azospirillum* sp. TSA6c and *Azospirillum doebereinerae* reflects the potential of the metagenome for metabolism of nitroaromatics, but more importantly the role this may play in assuaging the dearth of inorganic nutrient such as nitrogen in the FN1 soil. This is because the reductive process catalysed by this enzyme leads to release of nitrogen in the form of nitrite which can further be processed into ammonium and made available to the metabolizers and other organisms in the consortium [90].

It is interesting that fewer hydrocarbon degradation genes were annotated in the FN4 metagenome compared to FN1, which did not receive CFMM treatment. However, it suffices to note that this did not negatively impact the percentage degradation or components degraded at the end of 42 days. The plausible explanation for this might be that these genes encode enzymes with broad specificities for a wide range of pollutants. While some of these genes such at the ones for anthraniloyl-COA monooxygenase and N-ethylmaleimide reductase are the same as those annotated from the FN1 metagenome, others like those in some steps in the degradation of dioxin, xylene and benzoate were not preponderant in FN1. Dioxins are a common by-product of incomplete combustion and are known not only to be highly refractive, but also some of the most toxic and carcinogenic group of pollutants [91]. Some bacterial strains with dioxin and its analogue dibenzofuran degradation potentials were recently reported from polluted systems in Nigeria [92]. The enzymes 2-oxo-3-hexenedioate decarboxylase, 2-keto-4-pentenoate hydratase and 2-oxopent-4-enoate/cis-2-oxohex-4-enoate hydratase, which are involved in the metabolism of phenylalanine and phenolic compounds and *meta* cleavage of catechol [93], are widely distributed in the environment. These together with the genes annotated for salicylyl-COA 5-hydroxylase/oxidoreductase and 2-oxo-hepta-3-ene-1,7-dioic acid hydratase (*hpaH*) highlight the efficiency of the FN4 microcosm for the upscaling of the metabolism of naphthalene and other cocktail of aromatic compounds in the environment via pathways that were recruited over long period of perturbation [61, 94, 95].

The detection of the genes for biosurfactant production in the FN1 microcosm is not surprising because this is usually one of the adaptative responses of microorganisms in the environment to the low aqueous solubility and bioavailability of hydrocarbon compounds or resistance to heavy metals [96]. Biosurfactants are amphiphilic, low-molecular-weight microbial products, which dissolve in both polar and non-polar solvents and have surface activity that lowers the surface and interfacial tension between different phases [97, 98]. In recent times, metagenomic analysis has offered deeper insight into their role in hydrocarbon-polluted soil [99]. Rhamnolipid, one the biosurfactants whose genes were annotated in FN1, is a well characterized rhamnose sugar-containing surfactant almost exclusively produced by *Pseudomonas aeruginosa* while phosphatidylethanolamine on the other hand is a phospholipid produced mainly by *Rhodococcus* and other bacteria including Gram negatives [100, 101]*.* The role of these two classes of biosurfactants in oil emulsification and metal removal is well elucidated in literature [96]. The fact that the genes for biosurfactant were not annotated for the CFMM-treated FN4 microcosm is likely to be as a result of the dominant players utilizing other means for solubilization and accessing the hydrocarbon, more so that additional nutrient could favour the alternative mechanism over biosurfactant production which is known to depend not only on the metabolic capability of the organism but also the type of nutrient.

Some of the genes for bacterial chemotaxis proteins found in FN1 and FN4 such as *cheR*, *cheB*, *cheY*, *cheV* and *cheBR* had been previously detected in spent engine oil-polluted sites [50]. Chemotaxis is an important mechanism by which microorganisms negotiate their spatial relations with favourable or detrimental gradients in the environment and is known to be instrumental to access of some hydrocarbon degrading strains to nutrient sources [102–104]. Thus, the role of chemotaxis as a mechanism for adaptation in the animal charcoal-polluted soil appears not to be drastically affected by CFMM addition.

## Conclusion

Metagenomic analysis revealed a shift in microbial community structure with the order of predominance of phylotypes changing in the water-treated soil microcosm (FN1) from *Proteobacteria* (56.12 %) > *Actinobacteria* > *Firmicutes* to *Firmicutes* (92.97 %.) > *Proteobacteria* > *Actinobacteria* in carbon-free mineral medium-treated soil microcosm (FN4). This study also brought into prominence rarely isolated organisms that could further be explored for value-added products of biotechnology. There was also a remarkable reduction in the annotated genes in FN4. However, CFMM treatment resulted in about 10% increase in hydrocarbon degradation. The hydrocarbon degrading consortia in both microcosms were well adapted to overcoming the stressors in the environment. Water treatment and aeration without nutrient addition might be a cost-effective strategy for bioremediation of the animal charcoal site without concern for prolonged period of restoration to acceptable regulatory limit.

## Supplementary Information

**Additional file 1.**

## Data Availability

All data generated or analysed during this study are included in this published article and its supplementary information files

## Abbreviations

CFMM: Carbon-free mineral medium
GC-FID: Gas chromatography-flame ionization detector
STAMP: Statistical analysis of metagenomic profiles
HC: Hydrocarbon
