# Supplementary material for: Remarkable shift in structural and functional properties of an animal charcoal-polluted soil accentuated by inorganic nutrient amendment
Source: J Genet Eng Biotechnol. 2020 Nov 11;18:70. doi: 10.1186/s43141-020-00089-9 (PMC7658278; doi:10.1186/s43141-020-00089-9)
Supplement: Supplementary file 1 — Additional file 1. [file 43141_2020_89_MOESM1_ESM.docx]

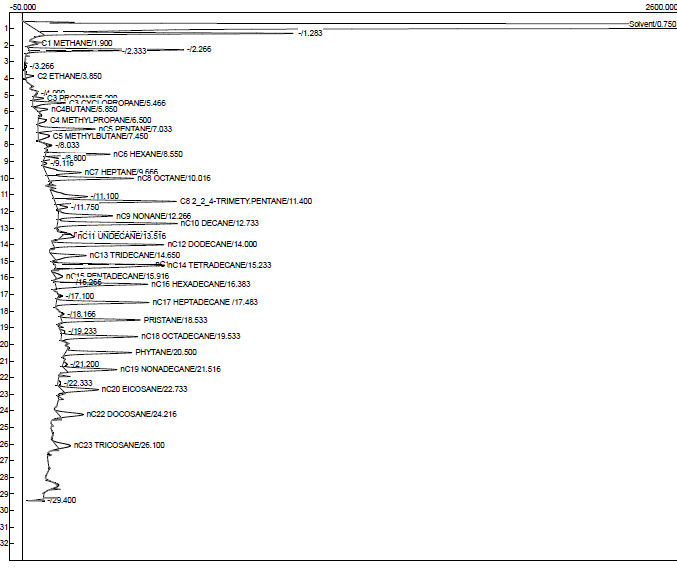


**a**


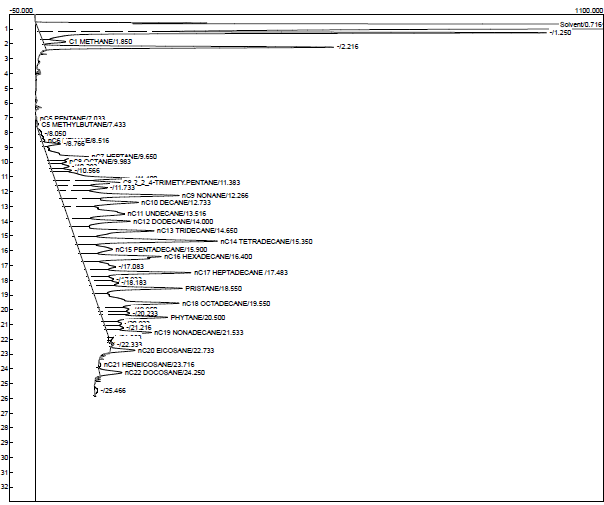


**b**


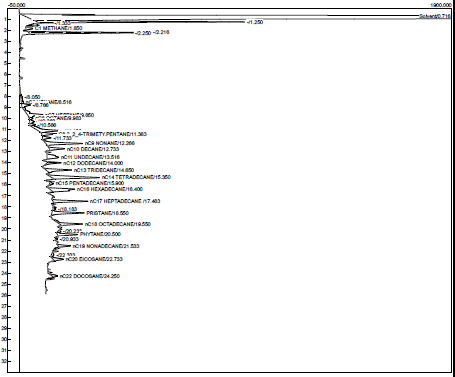


**c**

Figure S1: Gas chromatographic traces of n-hexane/acetone extract of aliphatic hydrocarbon fraction from FN1 soil microcosm at day 0 (a), day 21 (b), and day 42 (c) of incubation at room temperature. The oil components were separated on a 15 m long OV®-3 column (internal diameter, 0.53 mm; film thickness 0.25 μm)


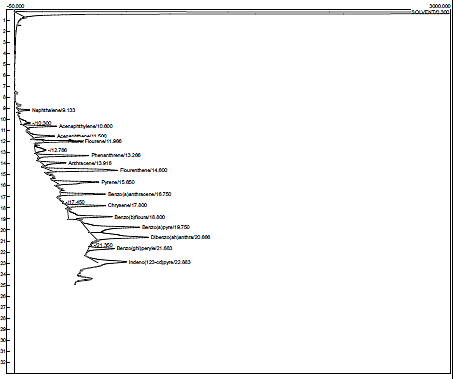


**a**


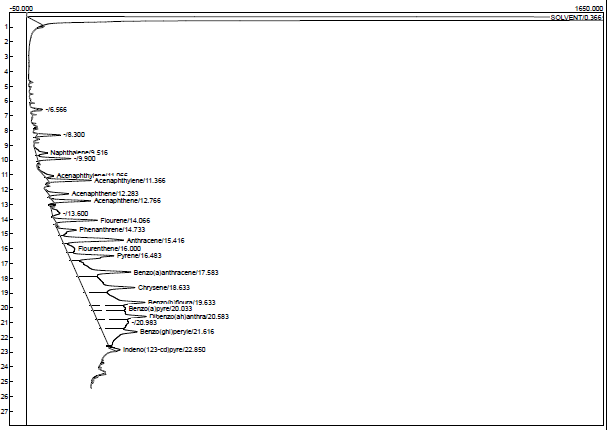


**b**


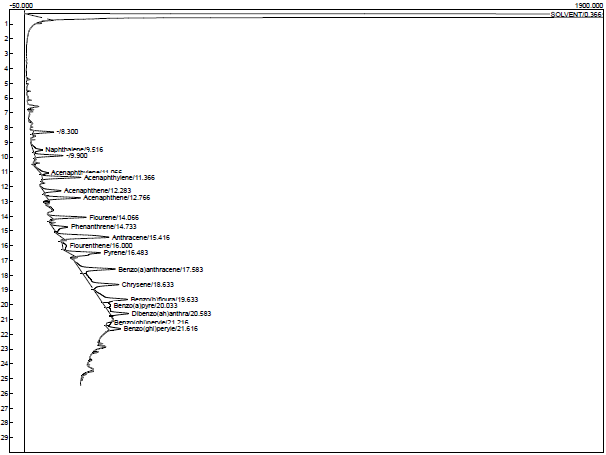


**c**

Figure S2: Gas chromatographic traces of n-hexane extract of aromatic hydrocarbon fraction from FN1 soil microcosm at day 0 (a), day 21 (b), and day 42 (c) of incubation at room temperature. The oil components were separated on a 15 m long OV®-3 column (internal diameter, 0.53 mm; film thickness 0.25 μm)


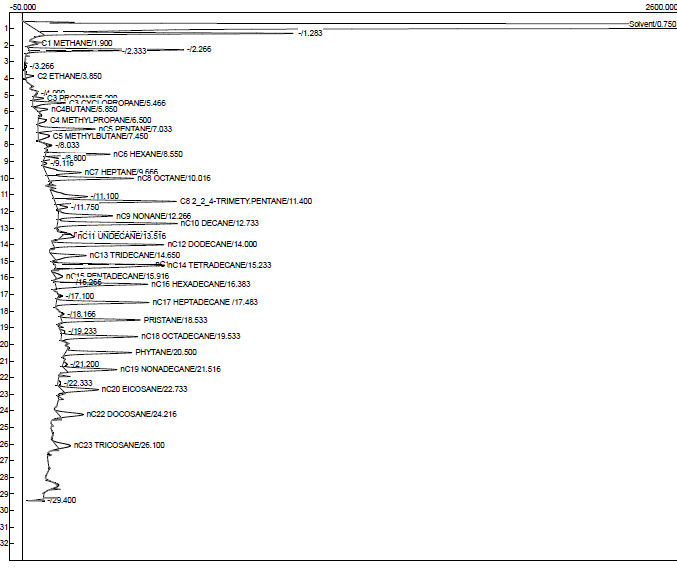


**a**


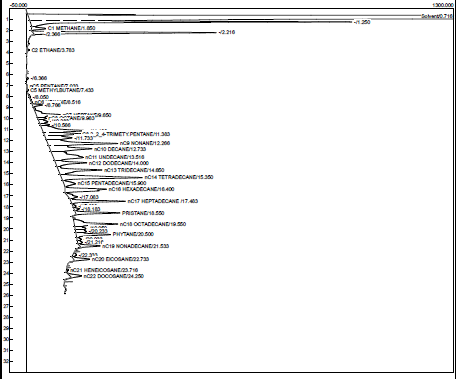


**b**


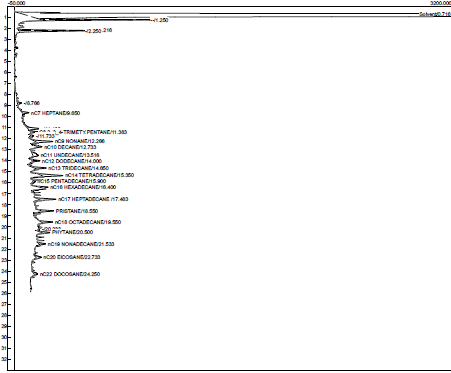


**c**

Figure S3: Gas chromatographic traces of n-hexane/acetone extract of aliphatic hydrocarbon fraction from FN4 soil microcosm at day 0 (a), day 21 (b), and day 42 (c) of incubation at room temperature. The oil components were separated on a 15 m long OV®-3 column (internal diameter, 0.53 mm; film thickness 0.25 μm)


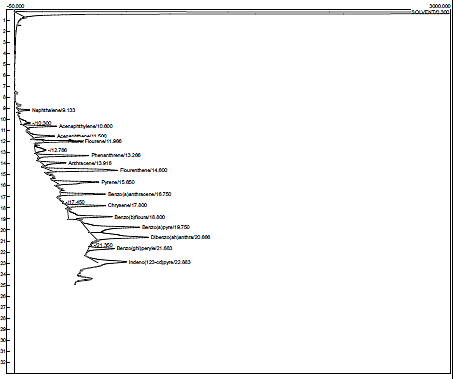


**a**


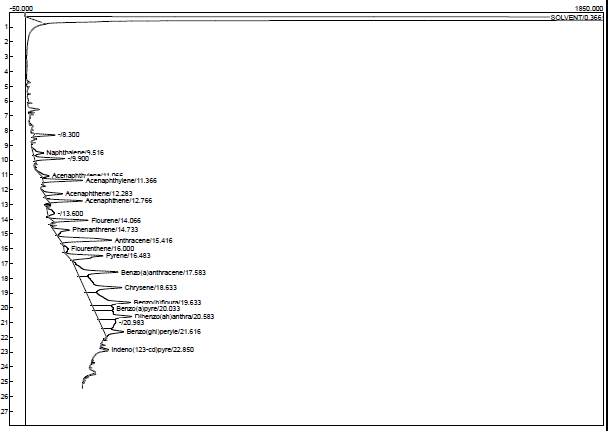


**b**


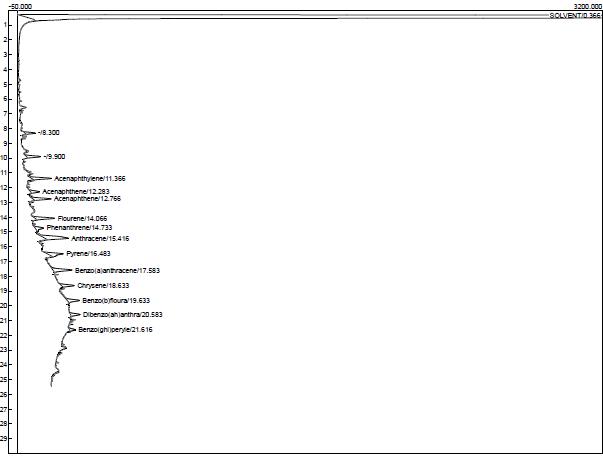


**c**

Figure S4: Gas chromatographic traces of n-hexane extract of aromatic hydrocarbon fraction from FN4 soil microcosm at day 0 (a), day 21 (b), and day 42 (c) of incubation at room temperature. The oil components were separated on a 15 m long OV®-3 column (internal diameter, 0.53 mm; film thickness 0.25 μm)


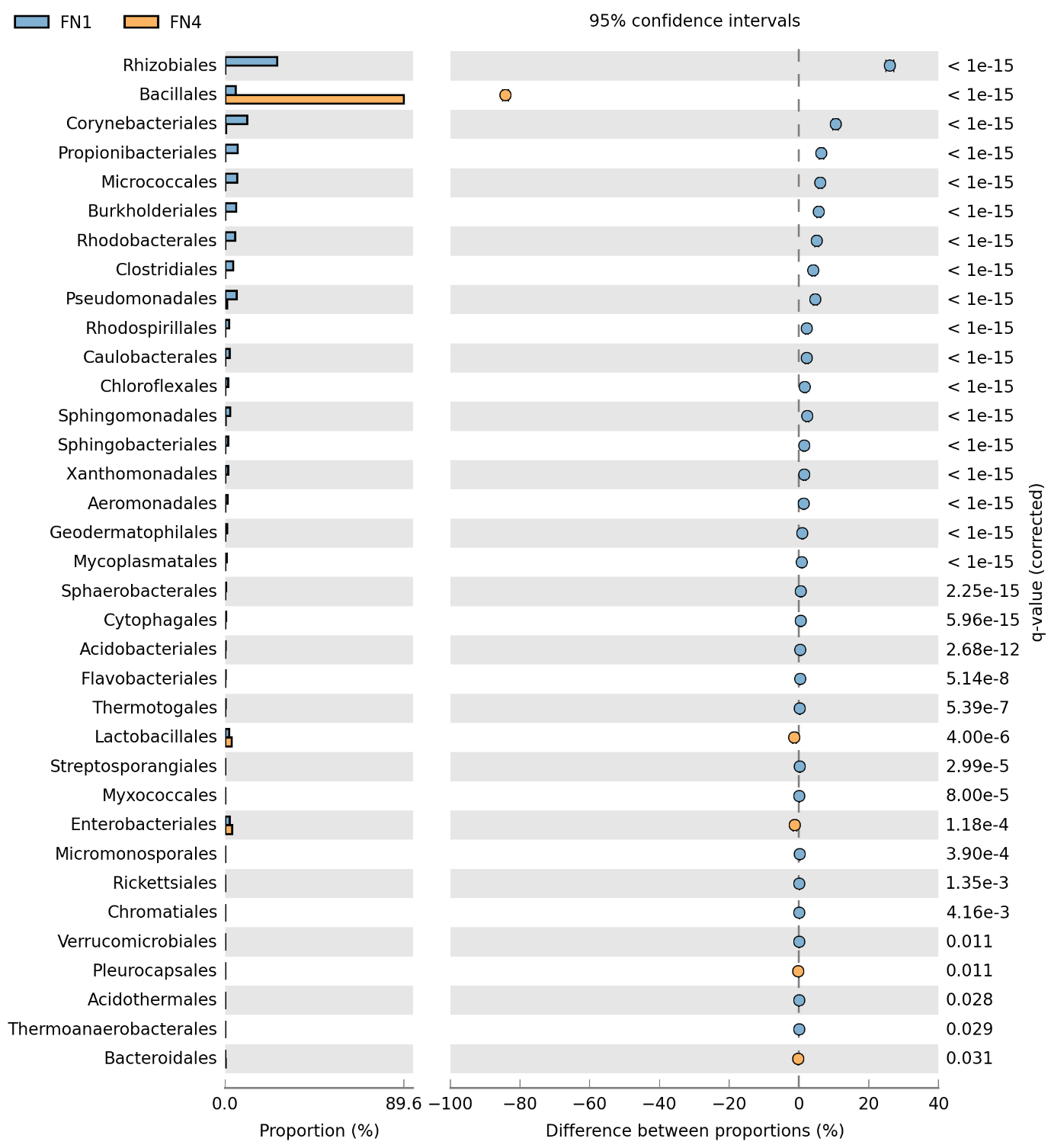


Figure S5: Comparative taxonomic profile of the FN1 and FN4 metagenomes at order delineation, computed by Kraken. Only orders with significant biological differences as determined by STAMP (P < 0.05, difference between the proportions >1% and twofold of ratio between the proportions) are shown.


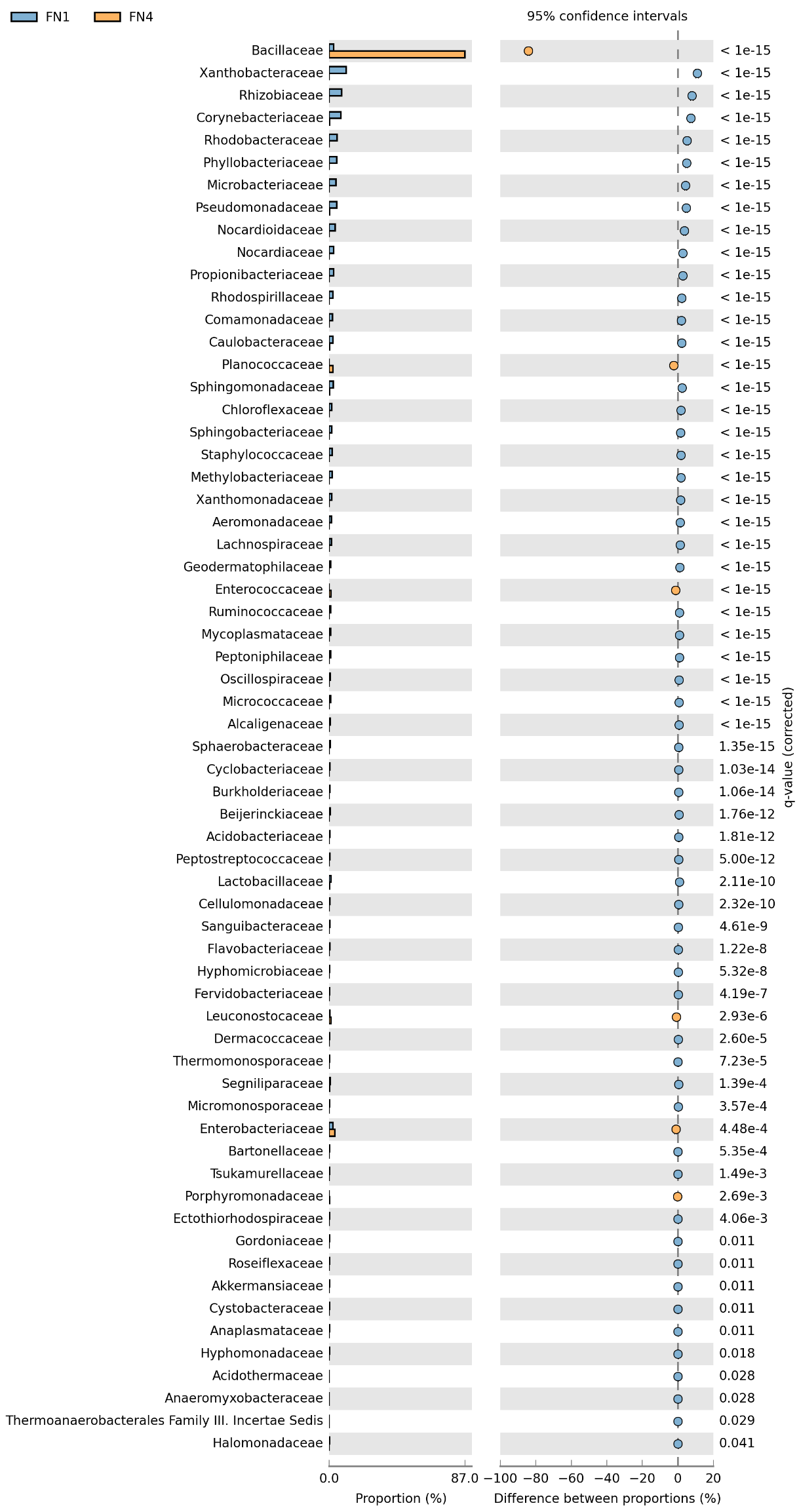


Figure S6: Comparative taxonomic profile of the FN1 and FN4 metagenomes at family delineation, computed by Kraken. Only families with significant biological differences as determined by STAMP (P < 0.05, difference between the proportions >1% and twofold of ratio between the proportions) are shown.

**REMARKABLE SHIFT IN STRUCTURAL AND FUNCTIONAL PROPERTIES OF AN ANIMAL CHARCOAL POLLUTED SOIL ACCENTUATED BY INORGANIC NUTRIENTS AMENDMENT**

**Abstract**

**Background**: Soils polluted with animal charcoal from skin and hides cottage industries harbour extremely toxic and carcinogenic hydrocarbon pollutants and thus require a bio-based eco-friendly strategy for their depuration. The effects of carbon-free mineral medium (CFMM) amendment on hydrocarbon degradation and microbial community structure and function in an animal charcoal polluted soil was monitored for six weeks in field moist microcosms consisting of CFMM treated soil (FN4) and an untreated control (FN1). Hydrocarbon degradation was monitored using gas chromatography-flame ionization detector (GC-FID), changes in microbial community structure was monitored using Kraken, while functional annotation of putative ORFs was done using KEGG KofamKOALA and NCBI’s conserved domain database (CDD). **Results**: Gas chromatographic analysis of hydrocarbon fractions revealed the removal 84.02% and 82.38% aliphatic and 70.09% and 70.14% aromatic fractions in FN4 and FN1 microcosms in 42 days. Shotgun metagenomic analysis of the two metagenomes revealed a remarkable shift in the microbial community structure. In FN4 metagenome, 92.97% of the population belong to the phylum *Firmicutes* and its dominant representative genera *Anoxybacillus* (64.58%), *Bacillus* (21.47%) and *Solibacillus* (2.39%). In untreated FN1 metagenome, the phyla *Proteobacteria* (56.12%), *Actinobacteria* (23.79%), and *Firmicutes* (11.20%); and the genera *Xanthobacter* (9.73%), *Rhizobium* (7.49%) and *Corynebacterium* (7.35%) were preponderant. Functional annotation of putative ORFs from the two metagenomes revealed the detection of degradation genes for aromatic hydrocarbons, benzoate, xylene, chlorocyclohexane/chlorobenzene, toluene, and several others in FN1 metagenome. In FN4 metagenome, only seven hydrocarbon degradation genes were detected. **Conclusion**: This study revealed that though CFMM amendment slightly increase the rate of hydrocarbon degradation, it negatively impacts the structural and functional properties of the animal charcoal polluted soil. It also revealed that intrinsic bioremediation of the polluted soil could be enhanced via addition of water and aeration.

**Keyword:** Animal charcoal polluted soil, Carbon-free mineral medium, Hydrocarbon degradation, Illumina shotgun sequencing, Microbial Community Structure, Soil microcosm

**Background**

The Nigerian environment is dotted by tens of thousands of sites polluted with animal charcoal. These are sites where animal hides, mainly from cow, are burnt or singed to remove the fur in the process of producing local delicacies called ‘Ponmo’.

The process involved often includes the use of kerosene, diesel, spent engine oil, plastics and old tyre as fuel [1]. It is poorly regulated and characterised by indiscriminate disposal of wastes, which are rich in animal charcoal laced with various types of hydrocarbons, including aliphatics, aromatics, polycyclic aromatic hydrocarbons (PAHs), dioxins, furans, benzenes and heavy metals [2, 4]. These wastes eventually find their way into surrounding soils, underground waters, runoffs, and surface waters. Recent report from a study of such site revealed hydrocarbon concentration far in excess of regulatory limit but very low concentration of inorganic nutrient [3, 4]. Since these sites are usually located around abattoirs and water canals or small streams, it is doubtless that this process could pose serious hazards to the ecosystem [5].

Hydrocarbons and heavy metal pollutants are known to pose health hazards resulting from toxicity, mutagenicity, teratogenicity and carcinogenicity [6, 7]. Although remediation efforts in the past focused more on physical and chemical approaches which are often very rapid. In the last few decades, attention has been directed to bioremediation or application of biological processes. This is mainly due to inherent shortcomings of the traditional processes, and the benign, effective, environmentally-friendly, and relatively inexpensive attributes of bioremediation approach [8]. In order to be able to implement an effective bioremediation programme, there is need for information on the pre-remedial physicochemistry and community diversity of the polluted system as well as monitoring of same during the bioremediation campaign [9, 15].

Furthermore, whereas, there is a plethora of reports on the characteristics of hydrocarbon polluted sites in Nigeria, there is presently dearth of information on the physicochemistry and microbial community diversity of majority of the animal charcoal polluted sites across the length and breadth of the country. Similarly, despite the presence of toxic and carcinogenic hydrocarbon pollutants in

animal charcoal polluted sites, there is paucity of efforts aimed at effective bioremediation of these sites. Availability of limiting nutrients such as phosphorus and nitrogen are pivotal for effective bioremediation of polluted sites. Biostimulation via addition of these limiting nutrients (organic or inorganic) have demonstrably enhanced hydrocarbon degradation [9, 15] While CFMM, rich in nitrogen and phosphorus sources have been used as an enrichment medium for isolation of hydrocarbon degraders [10], its use as a biostimulant to enhance hydrocarbon degradation in soil has not been established.

Culture-dependent approaches have been used to assess microbial community structure of polluted environments. However, the realization that it only reveals <1% of the members of the community makes it less desirable for microbial ecology studies [11]. The use of culture-independent approach such as shotgun metagenomics premised on sequencing the entire metagenome not only provide deep insights on the microbial ecology of contaminated sites but also give a snapshot of metabolic properties of members of the community [12, 13].

Here we report the dynamics of microbial community diversity and metabolic properties of a microcosm from a ‘ponmo’ cottage industry soil treated with carbon free mineral medium (CFMM). This is with a view to determining the efficiency of nutrient addition in reclamation and detection of novel genes and functionalities associated with animal charcoal contaminated systems. To the best of knowledge, this is the first report of shotgun metagenomic profiling of such sites.

**Methods**

**Sampling site description and soil microcosm set up**

Composite animal charcoal polluted soil samples were collected at an abattoir located in Ilorin, Kwara State, Nigeria. The sampling site, which has been in existence for 12 years, is where slaughtered animal hides and skin was burnt to remove the furs to produce a local Nigerian delicacy, “Ponmo”. The coordinates of the sampling site were latitude 8.471498 N and longitude 4.531245 E. Soil samples were collected at a depth of 10–12 cm with sterile hand trowel after clearing debris

from the soil surface. It was sieved using a 2-mm mesh size sieve. The sieved soil was thoroughly mixed in a large plastic bag to ensure homogeneity and was used without air-drying. The protocols for microcosm set up is as described by [15] with slight modifications. Sieved soil (1 kg) measured and placed in an open pan was designated FN1. The second soil microcosm designated FN4 contain 1 kg of sieved soil amended with 100 ml carbon-free mineral medium (CFMM; g L^-1^: NH_4_NO_3_, 3.0 g; Na_2_HPO_4_, 2.2 g; KH_2_PO_4_,0.8 g; MgSO4·7H_2_O, 0.1 g; FeCl_3_·6H_2_O, 0.05 g; and CaCl_2_·2H_2_O, 0.05 g; pH 7.0). The set ups (in triplicates) were incubated at room temperature (25 ± 3°C) for six weeks and flooded with 100 ml sterile distilled water to maintain a moisture level of 25%.

The physicochemistry and heavy metal content of the polluted soil were determined as described previously [4, 36]. The physicochemical properties indicated a pH of 5.51 ± 0.02, moisture content of 8.59% (± 0.16) and total organic content of 18.99 ± 0.18%. The nitrogen, phosphorus and potassium contents are 5.99 ± 0.06, 5.55 ± 0.12, and 12.99 ± 0.04 mg kg^-1^ soil, respectively. Similarly, background heavy metal content of the polluted soil revealed in mg kg^-1^ soil, the presence of iron (72.41 ± 0.72), lead (3.22 ± 0.11), zinc (12.50 ± 0.42, copper (8.99 ± 0.32), manganese (3.05 ± 0.01), cadmium (0.72 ± 0 .01), nickel (4.01 ± 0.01), and chromium (2.89 ± 0.02), respectively. Samples were taken from FN1 and FN4 microcosms for hydrocarbon content analyses at day 0, 21, and 42, respectively.

**Hydrocarbon content analysis of the microcosms**

Hydrocarbon content of the soil samples was determined by first drying the polluted soil samples (10 g) with 10 g anhydrous Na_2_SO_4_ in an extraction thimble. A mixture of analytical grade dichloromethane and acetone (10 ml; 1:1, v/v) was thereafter added and shaken for 30 min in a mechanical shaker. Collected samples was filtered into a glass beaker using a glass wool plugged into a glass funnel with 1 g anhydrous Na_2_SO4. The extraction was twice repeated and concentrated to 10 ml at 60 °C using rotary evaporator, after which 10 ml of hexane was added and further concentrated to about 1 ml at 60 °C. Clean-up and fractionation of the extract was done using silica gel permeation chromatography. Mixture of hexane and acetone (1:3 v/v; 10 ml) was used to extract the aliphatic fraction while 10 ml of *n*-hexane was used to extract the aromatic fraction. Residual

aliphatic and aromatic hydrocarbon fractions were determined by gas chromatography equipped with flame ionization detector (GC-FID) using OV^®^-3 column. The carrier gas is nitrogen. The injector and detector temperatures were maintained at 220 °C and 270 °C, respectively. The column was programmed at an initial temperature of50 °C for 2 min; ramped at 10°C/min to 250 °C and held for 5 min. The air flow, hydrogen flow and nitrogen flow rates are 450 ml min^−1^, 45 ml min^−1^ and 22 ml min^−1^, respectively.

**Total DNA extraction, shotgun metagenomics, processing of raw fastq reads, and read-based classification**

Total DNA used for metagenomic analysis was extracted directly from the two soil microcosms, FN1 and FN4. To discern the microbial community structure of the animal charcoal polluted soil prior to CFMM amendment, total DNA was extracted from the soil (FN1) immediately after sampling. For metagenomic evaluation of the effects of CFMM addition (100 ml kg^-1^ of soil) on the microbial community of the animal charcoal polluted soil, the total DNA was extracted from FN4 microcosm six weeks post CFMM addition. Total DNA were extracted from the sieved soil samples (0.25 g) using ZYMO soil DNA extraction Kit (Model D 6001, Zymo Research, USA) following manufacturer’s instructions. The quality and concentration of the extracted total DNA was ascertained using NanoDrop spectrophotometer and electrophoresed on a 0.9% (w/v) agarose gel, respectively. Shotgun metagenomics of FN1 and FN4 microcosms was prepared using the Illumina Nextera XT sample processing kit and sequenced on a MiSeq. The protocols for total DNA preparation for Illumina shotgun sequencing were as described previously [14, 15].

Pre-processing of fastq raw reads for quality profiling, read filtering, adapter trimming, quality filtering, polyG/polyX tail trimming, and per-read quality pruning was carried out using fastp, an ultra-fast FASTQ preprocessor [16]. The processed raw fastq reads was submitted to the Kraken taxonomic sequence classifier database [17] for read-based classification.

**Functional annotation of metagenomics reads**

Sequence reads generated from each of the metagenome were assembled individually using the make.contigs command in the MOTHUR metagenomic analysis suite [19]. Gene calling was performed on the assembled FN1 and FN4 contigs using MetaGene [20] to predict open reading frames (ORFs). The predicted genes (ORFs) were functionally annotated using the KEGG KofamOALA [21], which assigns K numbers to the predicted genes by HMMER/HMMSEARCH against KOfam (a customized HMM database of KEGG Orthologs), and the NCBI’s conserved domain database CDSEARCH/cdd v 3.15 (CDD) [22]. Taxonomic affiliation of the putative genes in the metagenomes was determined using AAI Profiler, which calculate average amino acid identity (AAI) between a query proteome (ORFs) and all target species in the Uniprot database [23].

**Accession Number**

The data, metadata and sequence reads of FN1 and FN4 metagenomes used in this study have been deposited in the European Nucleotide Archive (ENA) at EMBL-EBI under the accession number PRJEB37880 <https://www.ebi.ac.uk/ena/data/view/PRJEB37880>

**Statistical Analyses**

Statistical Analysis of Metagenomic Profiles, version 2 (STAMP) software [18] was used to statistically analysed the distinct taxonomic levels for each of the metagenomes retrieved from Kraken. Two-sided Fisher’s exact test with Newcombe–Wilson confidence interval method were used to determine the significance of the relative proportion difference in taxonomic distribution of FN1 and FN4 metagenomes, while Benjamini–Hochberg FDR was applied for correction. Unclassified reads were not used for analyses, and results with *q* < 0.05 were considered significant. The biological relevance of the statistic taxa was evaluated by applying a difference between the proportions of at least 1% and a twofold ratio between the proportions.

**Results**

**Kinetics of hydrocarbon degradation in FN1 and FN4 soil microcosms**

The degradation of aliphatic and aromatic hydrocarbons (HC) in FN1 and FN4 soil microcosms was monitored using GC/FID (Figure S1 and S2). In FN1 microcosm, the residual aliphatic HC content (554.98 mg/kg; 100%) decreased to 73.07% (405.51 mg/kg) after 21 days, corresponding to removal of 26.93% (149.47 mg/kg). At the end of 42 days, further decrease to 29.91% (165.98 mg/kg) in the residual aliphatic HC was observed, corresponding to removal of 70.09% (389 mg/kg) aliphatic HC (Figure S1). The residual aromatic HC content (471.56 mg/kg; 100%) decreased to 67.26% (317.19 mg/kg) after 21 days corresponding to the removal of 32.74% (154.37 mg/kg). The residual aromatic HC content decreased further at the end of 42 days, to 29.86% (140.81 mg/kg), corresponding to the removal of 70.14% (330.75 mg/kg) aromatic HC (Figure S2).

In FN4 microcosm, the residual aliphatic HC content (554.98 mg/kg; 100%) decreased to 51.27% (284.53 mg/kg) after 21 days, corresponding to removal of 48.73% (270.45 mg/kg). Further decrease in the residual aliphatic HC to 15.98% (88.66 mg/kg) was observed at the end of 42 days, corresponding to the removal of 84.02% (466.32 mg/kg) aliphatic HC (Figure S3). The residual aromatic HC content (471.56 mg/kg; 100%) decreased to 45.80% (215.98 mg/kg) after 21 days corresponding to the removal of 54.20% (255.58 mg/kg). Further decrease in the residual aromatic HC content to 17.62% (83.11 mg/kg) was observed after 42 days, corresponding to the removal of 82.38% (388.45 mg/kg) aromatic HC (Figure S4).

Significant changes in the degradation pattern of the hydrocarbon fractions were observed in FN1 and FN4 microcosms as shown in the GC fingerprints (Figures S1 and S2) and Table 1. In FN1 microcosm, the GC fingerprints of the aliphatic fractions showed complete disappearance of ethane, propane, cyclopropane, butane, methylpropane, pentane, methylbutane and tricosane fractions at the end of 42 days. Significant reductions of some fractions to <15% (hexane, octane, 2,2,4-trimethylpentane, decane, dodecane) and <30% (nonadecane, eicosane, docosane) were also observed. The GC fingerprint of the aromatic fractions revealed the disappearance of indeno(123-

cd)pyrene fractions, and significant reduction to <15% of fluoranthene, benzo(a)pyrene, and dibenzo(a)anthracene fractions, respectively (Figure S1, Table 1).

In FN4 microcosm, the GC fingerprints of the aliphatic fractions revealed complete disappearance of ethane, propane, cyclopropane, butane, methylpropane, pentane, methylbutane, hexane, octane and tricosane fractions at the end of 42 days. Apart from tetradecane, all the other aliphatic fractions were significantly reduced to <10% (2,2,4-trimethylpentane, decane, dodecane) and <30% (heptane, hexadecane, heptadecane, pristane, octadecane, phytane, nonadecane, eicosane, docosane) of their initial concentrations. The GC fingerprints of the aromatic fractions revealed complete disappearance of naphthalene, fluoranthene, benzo(a)pyrene and indeno(123-cd)pyrene fractions at the end of 42 days. Substantial reduction of some aromatic fractions to <10% (dibenzo(a)anthracene), <20% (phenanthrene, benzo(b)fluoranthene, benzo(ghi)perylene), and <30% (pyrene, chrysene) at the end of 42 days were also observed (Figure S2, Table 1).

**General characteristics of the metagenomes**

Illumina miseq shotgun sequencing of the two metagenomes resulted in 14,232 and 22,992 sequence reads for FN1 and FN4 with a total of 4,256,742 and 6,878,221 bp, a mean sequence length of 299 bp for both metagenomes, and a mean GC content of 57.31 and 57.72%, respectively. After pre-processing step with fastp, the sequence reads in FN1 and FN4 reduced to 13,500 and 21,624 with a total of 4,035,631 and 6,465,448 bp, the same mean sequence length and GC content of 57.22% for both metagenomes. The duplication rate in FN1 and FN4 sequence reads were 5.1 and 19.3%, while the insert size peak was 468 and 209, respectively. Additional information on the two metagenomes is available at <https://www.ebi.ac.uk/ena/data/view/PRJEB37880>

**Structural diversity of the metagenomes**

Analysis of the microbial community structure of the two metagenomes, FN1 and FN4 revealed significant differences in the taxonomic profiles generated by Kraken. In phylum classification where 14 and 8 phyla were recovered in FN1 and FN4 metagenome, the predominant phyla in FN1 are *Proteobacteria* (56.12%), *Actinobacteria* (23.79%), and *Firmicutes* (11.20%). In contrast, the

most dominant phylum in CFMM-amended FN4 metagenome was the *Firmicutes* with 92.97%. Other phyla with reasonable representation in FN4 were *Proteobacteria* (5.47%), and *Actinobacteria* (0.77%). Nine phyla, which are represented in FN1 metagenome completely disappeared in FN4, while the phyla *Planctomycetes*, *Cyanobacteria* and *Ignavibacteriae*, not represented in FN1 were duly detected in FN4 (Figure 1).

In class delineation, 22 and 11 classes were recovered from FN1 and FN4 metagenomes. The classes *Alphaproteobacteria* (40.61%), *Actinobacteria* (24.69%) and *Gammaproteobacteria* (11.08%) were preponderant in FN1, while *Bacilli* (93.11%) massively dominate in FN4, along with reasonable representation from the classes *Gammaproteobacteria* (4.74%) and *Actinobacteria* (0.77%). Thirteen classes, which were duly represented in FN1 completely disappeared in FN4, while the classes *Planctomycetia* and *Ignavibacteria* hitherto not present in FN1 were detected in FN4 (Figure 2).

Order classification revealed 53 and 28 orders in FN1 and FN4 metagenomes. The predominant orders in FN1 were *Rhizobiales* (26.30%), *Corynebacteriales* (11.06%), and *Propionibacteriales* (6.47%), while *Bacillales* (89.56%), *Enterobacteriales* (3.52%), and *Lactobacillales* (3.45%) were preponderant in FN4. Thirty orders, previously detected in FN1 completely disappeared in FN4 while the orders *Pleurocapsales*, *Planctomycetales*, *Ignavibacteriales*, *Desulfovibrionales*, and *Oscillatoriales* hitherto missing in FN1 were duly represented in FN4 (Figure S5).

Family delineation of FN1 and FN4 metagenomes revealed 99 and 54 families. The dominant families in FN1 were *Xanthobacteraceae* (10.94%), *Rhizobiaceae* (7.97%), and *Corynebacteriaceae* (7.59%), while in FN4, *Bacillaceae* (87.03%), *Enterobacteriaceae* (3.56%), and *Planococcaceae* (2.30%) were preponderant. Fifty-eight families previously detected in FN1 completely disappeared in FN4, while 13 families not detected in FN1 were duly represented in FN4 (Figure S6).

In genus classification, 155 and 95 genera were recovered from FN1 and FN4 metagenomes. The genera with highest representation in FN1 metagenome are *Xanthobacter* (9.73%), *Rhizobium*

(7.49%) and *Corynebacterium* (7.35%). In CFMM-amended FN4 metagenome, *Anoxybacillus* (64.58%), *Bacillus* (21.47%), and *Solibacillus* (2.39%) were preponderant. One-hundred and one (101) genera previously detected in FN1 metagenome completely disappeared in FN4, while 41 genera, hitherto not detected in FN1 were duly represented in FN4 metagenome (Figure 3).

**Functional characterization of the metagenomes**

Diverse hydrocarbon degradation genes were detected in FN1 metagenome as shown in Table 2. Putative genes responsible for degradation of benzoate (*pcaD, mhpF, aliB, benD-xylL, benC-xylZ, badH, had, dmpD, ligC, CMLE, pcaL, acd*, among others), xylene (*mhpF, benD-xylL, benC-xylZ, dmpD, cymB, cmtB*), chlorocyclohexane/chlorobenzene (*dehH, dhaA, linC, linX, pcpC*), and chloroalkane/chloroalkene (*adH, dehH, dhaA, adhP*) were detected. Also detected were degradative genes for toluene (*bbsG, bbsC, bbsD, tsaC1*), naphthalene (*adH, adhP, bnsG*), aminobenzoate (anthraniloyl-CoA monooxygenase, *ligC, lpdB*), ethylbenzene (*ped, etbD*), *dioxin* (*mhpF, bphD*), nitrotoluene (*nemA*), and several other aromatic hydrocarbons (Table 2). The benzoate and xylene degradation pathways, indicating the presence of the genes reported in this study and the reactions they catalysed in the pathways is depicted in Figure 4 and Figure 5. In FN4 metagenome, relatively few hydrocarbon degradation genes were detected. These include genes for 2-oxo-3-hexenedioate decarboxylase, 2-keto-4-pentenoate hydratase, and 2-oxopent-4-enoate/cis-2-oxohex-4-enoate hydratase involved in dioxin, xylene, and benzoate degradation. Others include putative genes for anthraniloyl-COA monooxygenase (aminobenzoate degradation), N-ethylmaleimide reductase (nitrotoluene degradation), bifunctional salicylyl-COA 5-hydroxylase/oxidoreductase (salicylate degradation), and 2-oxo-hepta-3-ene-1,7-dioic acid hydratase (*hpaH*) involved in 4-hydroxyphenylacetate and 2-oxopentenoate degradation.

Putative genes for uptake, transport, efflux and regulation of various inorganic nutrients and heavy metals were detected in FN1 metagenome (Table 3). These include genes for transport, uptake and regulation of phosphate/phosphonate (*pstB, pstS, phnC, phoB, ompR*), nitrogen (*urtD, urtE, nrtC, nrtD, glnC, ntrY, ntrX*), sufate/thiosulfate (*cysA, cysP, ssuB, sbp*) and several others. Putative genes for uptake, transport, efflux and regulation of heavy metals such as cobalt/nickel (*cbiO, nikD, nikE,*

*nrsR*), iron (*afuC, afuA, fhuC, fecE, fepC, fepA*), molybdate/tungstate (*modA, modB, modC, modF, tupC, wtpC*), manganese/zinc/iron (*znuC, psaB, mntB, mntA, sitB, troB, manR*), and copper (*nosF, cusR*) were also detected (Table 3). In FN4 metagenome, putative genes for transport, uptake and regulation of inorganic nutrients such as phosphate/phosphonate (*phoB, phnC, ompR*), and nitrogen (*narL, narP, ntrX, glnG*) were detected. For heavy metals, putative genes for transport, efflux and regulation such as *manR* (manganese), *cusR* (copper), *afuC* (iron) and *modC* (molybdate) were also detected.

Worth mentioning is the detection of genes responsible for biosynthesis of biosurfactants produced by members of the microbial community in FN1 metagenome. These include rhamnosyltransferase subunit B (*rhlB*), a member of *rhlAB* gene responsible for rhamnolipid biosynthesis, and phosphatidyl-N-methylethanolamine N-methyltransferase responsible for biosynthesis of a phospholipid biosurfactant, phosphatidylethanolamine It is instructive to note that these genes were not detected in FN4 metagenome. Putative genes responsible for bacterial chemotaxis were also detected from the two metagenomes. Putative genes for bacterial chemotaxis proteins *cheR, cheB, cheBR, cheY, aer, motB*, and *rbsB* were detected in FN1 while *cheB, cheY, cheV*, and *cheBR* were detected in FN4 metagenome.

**Discussion**

In order to be able to correctly assess the impact of a pollutant on an environmental compartment and proffer an appropriate strategy for reclamation, it is necessary to embark on a holistic and optimizable characterization of such site. Monitoring of trends in pollutant depletion and shifts in microbial community structures and functions relying especially on standard analytical chemistry methods and molecular approaches that enable access to both culturable and non-culturable phylotypes has gained currency as the best practice in this regard.

Analysis of the animal charcoal polluted soil revealed the presence of heavy metals, low inorganic nutrients, and an acidic pH. The acidic pH of the polluted system is a clear indication of an active catalytic activities of the autochthonous community on diverse hydrocarbons and organic materials

present in the soil, which yield acidic end products [24]. Although, in such acidic systems, there is a trade-off in diversity of phylotypes directly involved in the process as biodegradation of hydrocarbons by microorganisms has been shown to proceed relatively fast under conditions close to neutrality [4, 25].

In an actively-metabolizing microbial community, inorganic nutrients are always a limiting factor as they are required by members of the community for vital cellular functions and metabolic activities. This possibly explain the low concentrations of nitrogen and phosphorus in the polluted system. Functional annotation of the two metagenomes, FN1 and FN4 further revealed the limiting nature of these nutrients. For instance, the *pstB* and *pstS* are part of periplasmic transport system which are respectively ATP-binding and ATP-hydrolysis sites [26]. The *phnC* which was annotated for several species in the FN1 metagenome is encoded by the *phn* operon which is a member of the Pho regulon normally induced under phosphate starvation [27]. This suggests that these organisms were able to circumvent the shortage of inorganic phosphate in the polluted soil by activating the genes for acquisition and metabolism of less readily available organic sources requiring the activity of phosphate-carbon lyase. This is further buttressed by detection of two-component system genes *phoB* *ompR* and *cusR* which are part of the transduction systems that enable sense, respond and adapt to changes in their environment. While these genes are also annotated in FN4, it is only affiliated to *Klebsiella* sp., thus suggesting that phosphorus starvation is not prevalent in FN4, possibly due to addition of CFMM.

Similarly, while the two metagenomes, FN1 and FN4 were annotated for genes involved in inorganic nitrogen metabolism such as nitrate/nitrite transporter systems, the genes for urea transport were only annotated in FN1. This is interesting as the *urtD* and *urtE* genes are part of the genes normally activated in nitrogen limited environment for utilization of urea as nitrogen source and have been reported to be activated under such condition in some hydrocarbon degraders [28]. The absence of the *urtD* and *urtE* genes in FN4 could only be attributed to the addition of CFMM, which provide the needed inorganic nitrogen, thus making it impracticable due to energy costs to

use urea as nitrogen source. This possibly result in shutting down the metabolic pathway for urea transport and metabolism via feedback inhibition.

The detection of the putative genes for regulation, transport and efflux of heavy metals namely those for cobalt/nickel, molybdate/tungstate and manganese/zinc/iron underscore the importance of these metals both as stressors and as nutrient requirements of the microbial community. Heavy metals are common co-contaminants of hydrocarbon polluted sites arising as components of crude oil or acquired in the process of use, transport and disposal [29-31]. In excess of tolerance levels, heavy metals impose several inimical influences on microbial cells including cell membrane disruption, DNA damage, protein denaturation and inhibition of transcription, translation, enzyme activity and cell division. Several resistance mechanisms adopted to surmount this include internal and external sequestration, biosurfactant production, volatilization, precipitation and efflux pump systems [32-34]. It is equally noteworthy that most of the genes putatively identified in FN1 spanned various genera in the microcosm suggesting that they must have been acquired by and spread through the community horizontal gene transfer via mobile genetic elements [35-37].

It is noteworthy that the level of hydrocarbon pollution observed in the soil sample is more than the 500 mg/ kg limit established by regulatory bodies [3], although far less than the value of 2057.55 mg/kg we earlier reported for a similar site in Lagos, Nigeria [4]. In both FN1 and FN4 microcosms, there were considerable decreases in total hydrocarbon content during 42 days treatability period, indicating that there were autochthonous populations with hydrocarbon metabolism capability in the system. However, the fact that over 70 percent reduction in aliphatic and aromatic hydrocarbons was observed in FN1 where there was only addition of water and tilling highlights the important role of water activity as a limiting factor in the biodegradation of hydrocarbon pollutants in soil, especially in environment such as arid soil where moisture content is low or where water activity is low [38]. This trend has also been observed in our previous study [39]. Furthermore, it is not always the case that nutrient addition dramatically increases rate of degradation of hydrocarbons in soil. In some cases, nutrient addition had been reported to lead to insignificant or even negative outcomes [40, 41]. However, the initial more rapid rate of removal

of aliphatic and aromatic hydrocarbons in the FN4 microcosm in the first 21 days and subsequent overall higher total reduction is evidence of the stimulatory effect of the added CFMM.

The early disappearance of the lower-molecular-weight hydrocarbons, ethane, propane, cyclopropane, butane, methylpropane, pentane, methylbutane and tricosane in both microcosm and complete disappearance of hexane and octane in FN4 within the first 21 days is not surprising. These are low molecular-weight aliphatic fractions which are known to readily lend themselves to microbial degradation than the aromatic fractions (42, 43). However, it is not unlikely that some of the disappearance might be abiotic resulting from volatilization. The complete disappearance of all other aliphatics with a few exceptions, and complete disappearance of naphthalene, fluoranthene, benzo(a)pyrene and indeno(123-cd)pyrene in the FN4 after 42 days is noteworthy. This coupled with the fact that higher percentage removal was recorded for all other aromatic fractions including recalcitrant pyrene and chrysene in FN4 further demonstrated the role of nutrient amendment. Indeed, whereas the ability of bacteria in the environment to degrade the low molecular weight PAHs is wide spread, the same is not the case with fused four and five ring PAHs [44].

Metagenomic analysis showed remarkable difference in the diversity of the communities at the phyla level in FN1 and FN4, with the former having 14 phyla represented and the CFMM treated FN4 having representation in 8 phyla. The fact that the three most prominent phyla in FN1 are *Proteobacteria* (56.12%), *Actinobacteria* (23.79%), and *Firmicutes* (11.20%) is not surprising as previous reports had shown *Proteobacteria* and *Actinobacteria* to be the phyla more readily adapted to hydrocarbon polluted matrices, basically on account of their broad substrate specificity for diverse classes of hydrocarbons, and ability to survive in harsh, oligotrophic environments [45-51].

It is however interesting that many of the phyla which were represented in the FN1 soil completely disappeared in the FN4, leaving mainly the three mentioned above, with the *Firmicutes* accounting for more than 90 %. Though, the expectation was that nutrient addition would stimulate the dominant hydrocarbon degraders to blossom and express their functionality, it has been shown that bacteria richness and diversity in soil could be decreased when the soil is enriched with nitrogen

and phosphorus [52]. The dominant role of *Firmicutes* may not be unconnected with other hidden environmental factors that may limit the ability of the *Proteobacteria* and *Actinobacteria* to take advantage of the new nutrient regime. It is equally noteworthy that many *Actinobacteria* generally are oligotrophs and slow growers which can easily be overtaken by copiotrophs and are favoured in environment with pH 6 and 9 [53, 54]. Several authors have established that physicochemical parameters play a cardinal role in determining the community composition of hydrocarbon impacted systems and even a slight alteration in certain parameters could significantly skew the distribution in favour of a particular group [38, 55].

At the class level the *Alphaproteobacteria* dominated the FN1 microcosm followed by the *Actinobacteria* and *Gammaproteobacteria*. The two *Proteobacteria* classes are a group imbued with a variety of metabolic strategies, including nitrogen fixation, ammonia oxidation, chemoautotrophy, methylotrophy and temperature adaptation amongst others [56, 57]. It is therefore not unlikely that rather than nutrients such as nitrogen and phosphorus, water alone constituted the most important limiting factor. The preponderance of *Rhizobiales* (26.30%), at the order level and the *Xanthobacteriaceae* (10.94%) and *Rhizobiaceae* (7.9 %) at the family level seem to further buttress this, as the prominence of these groups in ecosystem where available nutrients were depleted as a result of hydrocarbon contamination has been reported [58]. The predominant phenotypes at genus level namely, *Xanthobacter* (9.73%), *Rhizobium* (7.49%) and *Corynebacterium* (7.35%) have strains which have been well reported in the biodegradation of both aliphatic and aromatic fractions in hydrocarbon polluted soils [59-61].

The two most represented genera in the CFMM-amended FN4 microcosm were *Anoxybacillus* (64.58%) and *Bacillus* (21.47%) both of which are members of the family *Bacillaceae* in the order *Bacillales*. They are both endospore-forming Gram-positive rods. Predominance of the *Anoxybacillus* in FN4 is of interest because species of this unique genus have been isolated from diverse extreme environments including anoxic, geothermal springs and heavy metal rich systems [62-65]. Although mostly alkali-tolerant thermophiles, they have been found to be physiologically diverse with respect to optimal temperature of growth and pH, with some being aerobic and others

anaerobic [63]. While the genus *Anoxybacillus* are not recognised as a prominent player in the degradation of hydrocarbons, Al-Jailawi et al. [66] reported a thermophilic *Anoxybacillus rupeinsis* strain Ir3 isolated from a hydrocarbon polluted site in Iran with metabolic propensity for aromatic compounds including carbazole, ρ-nitrophenol, nitrobenzene and naphthalene. The up scaling of *Anoxybacillus* as the predominant genus in the CFMM treated animal charcoal polluted soil FN4 may not be unconnected to the fact that the soil represented a localized extreme environment in which most other potential hydrocarbon degraders were not favoured. Furthermore, it is also not unlikely that *Anoxybacillus* strains were able to take advantage of the nutrient addition than their potential rivals.

*Bacillus* is the largest and best studied genus in terms of the number of isolates, studies conducted and published reports among the *Bacillaceae* [67, 68]. It is a long-standing genus in terms of hydrocarbon degradation capability and reports abound in the literature of its metabolic capabilities on saturated, aromatic and heteroaromatic hydrocarbon fractions [69]. Therefore, detection of *Bacillus* as the second most represented genus in the FN4 microcosm is not entirely surprising. *Solibacillus*, which is the third (2.39%) most preponderant genus in the FN4 soil, is a small group in the family *Planococcaceae*. Only three rarely isolated species are known, namely *S. silvestris, S. isorensis* [70, 71] and *S. kalamii* [72]. Members of the genus have been isolated from cryotube used for sampling the upper atmosphere, high-efficiency particulate arrestance (HEPA) filter, and identified as key colonizers of biofilter in a waste gas treatment plant [72, 73]. Evidently, the *Solibacillus* appear to be organisms adapted to high-stressed environments [74]. It is therefore not surprising that they are making an impressive appearance in such a unique and little understood ecosystem as the soil of cottage industry producing Ponmo. Thus, the biostimulation with CFMM dramatically altered the community structure in favour of unique, rarely isolated and physiologically versatile strains, which deserve to be further explored by isolating and properly characterizing them.

Hydrocarbon polluted sites is usually co-contaminated with other xenobiotic compounds and heavy metals. This usually triggers avalanche of responses from the autochthonous microbial community

to counteract the negative impacts of the environmental stressors. The metabolic functions highlighted in the genes identified in FN1 metagenome not only reaffirm the cocktail of pollutants with which the animal charcoal site is laced alongside the hydrocarbons but is also a reflection of the metabolism of these pollutants by the resident flora [75]. It is equally not surprising that the groups of organisms belonging to the order *Rhizobales* (particularly *Rhizobium* and *Mesorhizobium*) and *Actinobacteria* which were the predominant taxa were also the ones annotated for the metabolic functions.

The rapid disappearance of short-chained aliphatics in FN1 microcosm may not be unconnected to activity of alcohol dehydrogenase, which was detected in this study. The alcohol dehydrogenase catalyzed step is a very important one in the metabolic processing of aliphatic hydrocarbons and side chain reactions of aromatics, and their derivatives. The enzyme has a broad substrate specificity on various classes of aliphatic, alicyclic, and aromatic alcohols and hydroxyls [76-79].

The initial step in the aerobic degradation of aromatic compound usually involves the dihydroxylation of one of the polynuclear aromatic rings by incorporation of two atoms of oxygen into the aromatic ring. This process activates the molecule compromising its integrity as it prepares it for cleavage. Ring hydroxylation is catalysed by a multi component dioxygenase which consists of a reductive, a ferredoxin, and an iron sulphur protein, while ring cleavage is generally catalysed by an iron containing *meta* cleavage enzyme. The carbon skeleton produced by the ring cleavage reaction is then dismantled, before cleavage of the second aromatic ring [80]. The subsequent steps involve a number of enzymes which catalyze a range of reactions including hydroxylation and cleavage and eventual processing through either an *ortho* or a *meta* cleavage type of pathway, leading to central intermediates such as protocatechuate and catechol, which are further converted to tricarboxylic acid cycle intermediate [81, 82].

In this study, many of the annotated genes encoding enzymes (dehydrogenases, dioxygenase-reductases, hydrolases, decarboxylases, lactonases) that channelled the metabolic intermediates resulting from degradation of aromatics, particularly benzoates, xylene, toluene, naphthalene, and ethylbenzene into the tricarboxylic acid were affiliated to the *Rhizobales* (*Mesorhizobium*) and

*Nocardioides*. Thus, it appears that while other players no doubt contributed to the degradation of these compounds; the aforementioned genera must have recruited overtime most of the genetic capability for utilization of these aromatic compounds. Of interest are the genes annotated for benzoate degradation in *Nocardioides*, namely cyclohexanecarboxyl-CoA dehydrogenase, pimeloyl-CoA dehydrogenase, glutaryl-CoA dehydrogenase (non-decarboxylating), cyclohex-1-ene-1-carbonyl-CoA dehydrogenase, cyclohexane-1-carbonyl-CoA dehydrogenase and those annotated for *Candidatus Rokubacteria,* *Microbacterium* and *Rhizobales* namely dihydroxycyclohexadiene carboxylate dehydrogenase (*benD-xylL*); 2-hydroxycyclohexanecarboxyl-CoA (*badH*) and 6-hydroxycyclohex-1-ene-1-carbonyl-CoA dehydrogenase (*had*). These are enzymes involved in the syntrophic production of cyclohexane carboxylate and acetate in the presence of benzoate [83, 84], which suggests that syntrophism, in which *Actinobacteria* such as *Nocardioides, Microbacterium* and *Candidatus Rokubacteria* are key symbionts is an important interaction that facilitate pollutant removal in the FN1 animal charcoal microcosm.

The annotation of the genes encoding haloacetate dehalogenase (*DehH*) , haloalkane dehalogenase (*dhaA*), 2,5-dichloro-2,5-cyclohexadiene-1,4-diol dehydrogenase 1 (*linC*) and 2,5-dichloro-2,5-cyclohexadiene-1,4-diol dehydrogenase 2 (*linX*) in relation to chlorocyclohexane, chlorobenzene, chloroalkane and chloroalkene metabolism is not surprising as the dehalogenation steps are usually important in the detoxification and ultimate mineralization of halogenated hydrocarbons. This group of enzymes catalyzes the cleavage of the carbon-halogen bond of organohalogen compounds and has potential applications in the chemical industry and bioremediation. Hydrolytic dehalogenation is commonly performed by haloalkane dehalogenase, 2-haloacid dehalogenase, and 4-chlorobenzoyl-CoA dehalogenase, while reductive respiratory dehalogenation is carried out by organisms which derive energy from the dehalogenation process [85, 86]. Haloalkane dehalogenases convert haloalkanes to their corresponding alkanes, halides and protons [87, 88]. These genes, like most genes associated with the degradation of xenobiotics are acquired by horizontal transfer and localized on mobile genetic elements like plasmids over time. Presence of

these genes in the metagenome suggests chronic contamination of the site with halogenated compounds.

Genes encoding some steps in the degradation of aminobenzoate were also annotated in the FN1 metagenome including anthraniloyl-CoA monooxygenase, 2-hydroxy-4-carboxymuconate semialdehyde hemiacetal dehydrogenase (*ligC*) and gallate decarboxylase subunit B (*lpdB*). 2-Aminobenzoate is a derivative of tryptophan and other indole containing compounds. It is one of the intermediates in the degradation of *orho* nitrobenzoate and carbazole. One way apart from direct processing to catechol, by which it is aerobically metabolized is the activation to 2-Aminobenzoyl-CoA by Anthranilate CoA ligase. The oxidoreductase enzyme Anthraniloyl CoA monooxygenase oxidizes this into 2-Amino-5-oxo-cyclohex-1-enecarboxy-CoA [89]. Similarly, the annotation of *nemA* - N-ethylmaleimide reductase affiliated with *Azospirillum* sp. TSA6c and *Azospirillum doebereinerae* reflects the potential of the metagenome for metabolism of nitroaromatics, but more importantly the role this may play in assuaging the dearth of inorganic nutrient such as nitrogen in the FN1 soil. This is because the reductive process catalysed by this enzyme leads to release of nitrogen in the form of nitrite which can further be processed into ammonium and made available to the metabolizers and other organisms in the consortium [90].

It is interesting that fewer hydrocarbon degradation genes were annotated in the FN4 metagenome compared to FN1, which did not receive CFMM treatment. However, it suffices to note that this did not negatively impact the percentage degradation or components degraded at the end of 42 days. The plausible explanation for this might be that these genes encode enzymes with broad specificities for wide range of pollutants. While some of these genes such at the ones for anthraniloyl-COA monooxygenase and N-ethylmaleimide reductase are the same as those annotated from the FN1 metagenome, others like those in some steps in the degradation of dioxin, xylene, and benzoate were not preponderant in FN1. Dioxins are a common by-product of incomplete combustion and are known not only to be highly refractive, but also some of the most toxic and carcinogenic group of pollutants [91]. Some bacterial strains with dioxin and its analogue dibenzofuran degradation potentials were recently reported from polluted systems in Nigeria [92].

The enzymes 2-oxo-3-hexenedioate decarboxylase, 2-keto-4-pentenoate hydratase, and 2-oxopent-4-enoate/cis-2-oxohex-4-enoate hydratase, which are involved in the metabolism of phenylalanine and phenolic compounds and *meta* cleavage of catechol [93] are widely distributed in the environment. These together with the genes annotated for salicylyl-COA 5-hydroxylase/oxidoreductase, and 2-oxo-hepta-3-ene-1,7-dioic acid hydratase (*hpaH*) highlight the efficiency of the FN4 microcosm for the up scaling of the metabolism of naphthalene and other cocktail of aromatic compounds in the environment via pathways that were recruited over long period of perturbation [61, 94, 95].

The detection of the genes for biosurfactant production in the FN1 microcosm is not surprising because this is usually one of the adaptative responses of microorganisms in the environment to the low aqueous solubility and bioavailability of hydrocarbon compounds or resistance to heavy metals [96]. Biosurfactants are amphiphilic, low molecular weight microbial products, which dissolve in both polar and non-polar solvents and have surface activity that lowers the surface and interfacial tension between different phases [97, 98]. In recent times, metagenomic analysis has offered deeper insight into their role in hydrocarbon polluted soil [99]. Rhamnolipid, one the biosurfactants whose genes were annotated in FN1, is a well characterised rhamnose sugar containing surfactant almost exclusively produced by *Pseudomonas aeruginosa* while phosphatidylethanolamine on the other hand is a phospholipid produced mainly by *Rhodococcus* and other bacteria including Gram negatives [100, 101]*.* The role of these two classes of biosurfactants in oil emulsification and metal removal is well elucidated in literature [96]. The fact that the genes for biosurfactant were not annotated for the CFMM-treated FN4 microcosm is likely to be as a result of the dominant players utilizing other means for solubilisation and accessing the hydrocarbon, more so that additional nutrient could favour the alternative mechanism over biosurfactant production which is known to depend not only on the metabolic capability of the organism but also the type of nutrient.

Some of the genes for bacterial chemotaxis proteins found in FN1 and FN4 such as *cheR, cheB, cheY, cheV*, and *cheBR* had been previously detected in spent engine oil polluted sites [50]. Chemotaxis is an important mechanism by which microorganisms negotiate their spatial relations

with favorable or detrimental gradients in the environment and is known to be instrumental to access of some hydrocarbon degrading strains to nutrient sources [102-104]. Thus, the role of chemotaxis as a mechanism for adaptation in the animal charcoal polluted soil appears not to be drastically affected by CFMM addition.

**Conclusion**

Metagenomic analysis revealed a shift in microbial community structure with the order of predominance of phylotypes changing in the water treated soil microcosm (FN1) from *Proteobacteria* (56.12 %) > *Actinobacteria* > *Firmicutes* to *Firmicutes* (92.97 %.) > *Proteobacteria* > *Actinobacteria* in carbon free mineral medium treated soil microcosm (FN4). This study also brought into prominence rarely isolated organisms that could further be explored for value added products of biotechnology. There was also a remarkable reduction in the annotated genes in FN4. However, CFMM treatment resulted in about 10% increase in hydrocarbon degradation. The hydrocarbon degrading consortia in both microcosms were well adapted to overcoming the stressors in the environment. Water treatment and aeration without nutrient addition might be a cost-effective strategy for bioremediation of the animal charcoal site without concern for prolonged period of restoration to acceptable regulatory limit.

**List of Abbreviations**:

CFMM: Carbon-Free Mineral Medium

GC-FID: Gas Chromatography-Flame Ionization Detector

STAMP: Statistical Analysis of Metagenomic Profiles

HC: Hydrocarbon

**References**

1. Akwetey WY, Eremong DC, Donkoh A (2013) Chemical and nutrient composition of cattle hide (“Welle”) using different processing methods. J Animal Sci Adv 3(4):176-180.
2. Oko OJ, Okoye COB (2017) Quantification of smoke contributed PAHs in roasted cowhide (ponmo) from northern Nigeria. FUW Trends Sci Technol J 2(1A):55-59.
3. DPR (1991) Guidelines and Standards for the Petroleum Industry in Nigeria. Department of Petroleum Resources, Lagos, Nigeria.
4. Obayori OS, Salam LB, Oyetibo GO, Idowu M, Amund OO (2017) Biodegradation potentials of polyaromatic hydrocarbon (pyrene and phenanthrene) by *Proteus mirabilis* isolated from an animal charcoal polluted site. Biocatalysis Agric Biotechnol 12:78–84.
5. Nguyen T, Hlangothi D, Martinez 3rd RA, Jacob D, Anthony K, Nance H, Saleh MA (2013) Charcoal burning as a source of polyaromatic hydrocarbons in waterpipe smoking. J Environ Sci Health B 48:1097–1102.
6. Domingo JL, Nadal M (2015) Human dietary exposure to polycyclic aromatic hydrocarbons: a review of the scientific literature. Food Chem Toxicol 86:144–153.
7. Abdel-Shafy, HI, Mansour MSM (2016) A review on polycyclic aromatic hydrocarbons: source, environmental impact, effect on human health and remediation. Egypt J Pet 25: 107–123.
8. Abatenh E, Gizaw B, Tsegaye Z, Wassie M (2017) Application of microorganisms in bioremediation-review. J Environ Microbiol 1(1):02-09.
9. Chikere CB, Okpokwasili GC, Chikere BO (2011) Monitoring of microbial hydrocarbon remediation in the soil. 3 Biotech 1:117-138.
10. Salam LB, Ilori MO, Amund OO, LiiMien Y, Nojiri H (2018) Characterization of bacterial community structure in a hydrocarbon-contaminated tropical African soil. Environ Technol 39(7):939-951.
11. Sabale SN, Suryawanshi P, Krishnayaj PU (2019) Metagenomics – basic methods and applications. In: Soil metagenomics: Concepts and applications. *DOI:* <http://dx.org/10.5772/intechopen.88958>.
12. Jeffries TC, Rayu S, Nielsen UN, Lai K, Ijaz A, Nazaries L, Singh BK (2018) Metagenomic functional potential predicts degradation rates of a model organophosphorus xenobiotic in pesticide contaminated soil. Front Microbiol [http://dx.org/10.3389/fmicb .2018.00147](http://dx.org/10.3389/fmicb%20.2018.00147).
13. Salam LB (2019) Metagenomic insights into the diversity and functions of microbial assemblages in lakes. In: Bandh SA, Shafi S, Shameem N (eds.), Freshwater Microbiology: Perspectives of bacterial dynamics in lake ecosystems. First Edition, Academic Press, an imprint of Elsevier, London, United Kingdom, pp. 175- 210.
14. Salam LB (2018) Detection of carbohydrate active enzymes and genes in a spent engine oil perturbed agricultural soil. Bull Nat Res Centre 42:10.
15. Salam LB, Ishaq A (2019) Biostimulation potentials of corn steep liquor in enhanced hydrocarbon degradation in chronically polluted soil. 3 Biotech 9:1-20.
16. Chen S, Zhou Y, Chen Y, Gu J (2018) Fastp: an ultra-fast all-in-one FASTQ preprocessor. Bioinformatics 34(17):i884-i890.
17. Wood DE, Salzberg SL (2014) Kraken: ultrafast metagenomic sequence classification using exact alignments. Genome Biol 15(3):R46.
18. Parks DH, Tyson GW, Hugenhiltz P, Beiko RG (2014) STAMP: statistical analysis of taxonomic and functional profiles. Bioinformatics 30(21):3123–3124.
19. Schloss PD, Westcott SL, Ryabin T et al (2009) Introducing mothur: open-source, platform-independent, community-supported software for describing and comparing microbial communities. Appl Environ Microbiol 75(23):7537-7541.
20. Noguchi H, Park J, Takagi T (2006) MetaGene: prokaryotic gene finding from environmental genome shotgun sequences. Nucleic Acids Res 34(19):5623–5630.
21. Aramaki T, Blanc-Mathieu R, Endo H, Ohkubo K, Kanehisa M, Goto S, Ogata H (2019) KofamKOALA: KEGG ortholog assignment based on profile HMM and adaptive score threshold. Bioinformatics Btz859.
22. Marchler-Bauer A, Derbyshire MK, Gonzales NR et al (2015) CDD: NCBI’s conserved domain database. Nucleic Acids Res 43(D):222–226.
23. Medlar AJ, Törönen P, Holm L (2018) AAI-profiler: fast proteome-wide exploratory analysis reveals taxonomic identity, misclassification and contamination. Nucleic Acids Res 46(W1):W479-W485.
24. Ilori MO, Amund OO, Obayori OS, Omotayo AE (2015) Microbial population and physico-chemical dynamics of a soil ecosystem upon petroleum contamination. J Sci Res Dev 15:25–33.
25. Vidali M (2001) Bioremediation. An overview. Pure Appl Chem 73:1163–1172.
26. McGrath JW, Chin JP, Quinn JP (2013) Organophosphonates revealed: new insights into the microbial metabolism of ancient molecules. Nat Rev Microbiol 11:412–419. <http://dx.doi.org/10.1038/nrmicro3011>.
27. Stosiek N, Talma M, Klimek-Ochab M (2020) Carbon-Phosphorus Lyase—the State of the Art. Appl Biochem Biotechnol 190:1525–1552.
28. Tikariha H, Purohit HJ (2019) Assembling a genome for novel nitrogen-fixing bacteria with capabilities for utilization of aromatic hydrocarbons. Genomics 111:1824-1830.
29. Dominguez-Rusado E, Pitchel J (2003) Chemical characterization of fresh and weathered motor oil via GC/MS, NMR and FITR Techniques. Proceedings of the Indiana Academy of Science 112:109-116.
30. Osuji LC, Onojake CM (2004) Trace heavy metals associated with crude oil: a case study of ebocha-8 oil-spill-polluted site in Niger Delta, Nigeria. Chem Biodivers 1:1708–1715.
31. Oyetibo GO, Chien M-F, Ikeda-Ohtsubo W, Suzuki H, Obayori OS, Adebusoye SA, Ilori MO, Amund OO, Endo G (2017) Biodegradation of crude oil and phenanthrene by heavy metals resistant *Bacillus subtilis* isolated from multi-polluted industrial wastewater creek. Int Biodeterior Biodegrad 120:143-151.
32. Outten FW, Outten CE, O’Halloran T (2000) Metalloregulatory systems at the interface between bacterial metal homeostasis and resistance. In: Storz G, Hengge-Aronis R (eds) Bacterial stress responses. ASM Press, Washington D.C, pp 145–157.
33. Roane TM, Rensing C, Pepper L, Maier RM (2009) Microorganisms and Metals. In: Environmental Microbiology, Miaer RM, Piepper IL, Gerba CP (eds), Academic Press, Burlington, pp. 421-441.
34. Oyetibo GO, Ilori MO, Adebusoye SA, Obayori OS, Amund OO (2010) Bacteria with dual resistance to elevated concentrations of heavy metals and antibiotics in Nigerian contaminated systems. Environ Monit Assess 168:305–314.
35. Li L-G, Zhang T (2015) Exploring antibiotic resistance genes and metal resistance genes in plasmid metagenomes from wastewater treatment plants. Front Microbiol 6:1025. doi:10.3389/fmicb.2015.01025.
36. Salam LB, Obayori OS, Ilori MO, Amund OO (2020) Effects of cadmium perturbation on the microbial community structure and heavy metal resistome of a tropical agricultural soil. Bioresour Bioprocess 7:25.
37. Veress A, Nagy T, Wilk T, Komuves J, Olasz F, Kiss Jl (2020) Abundance of mobile genetic elements in an *Acinetobacter iwoffii* strain isolated from Transylvanian honey sample. Sci Rep 10:2969 <https://doi.org/10.1038/s41598-020-59938-9>.
38. Gao S, Liang J, Teng T, Zhang M (2019) Petroleum contamination evaluation and bacterial community distribution in a historic oilfield located in loess plateau in Cina. Appl Soil Ecol 136:30-42.
39. Salam LB, Idris H (2019) Consequences of crude oil contamination on the structure and function of allochthonous microbial community of a tropical agricultural soil. Environ Sustain 2(2):167-187.
40. Carmichael LM, Pfaender FK (1997) The effects of inorganic and organic supplements on the microbial degradation of phenanthrene and pyrene in soils. Biodegradation 8:1–13.
41. Chaineau CH, Rougeux G, Yepremain C (2005) Effects of nutrients concentrations on the biodegradation of crude oil and associated microbial populations in the soil. Soil Biol Biochem 37:1490–1497.
42. Philp JC, Whiteley AS, Ciric L, Bailey MJ (2005) Monitoring bioremediation In: Atlas RM, Philp J (eds.). Bioremediation: Applied Solution for a Real-World Environmental Clean Up. ASM Press, Washington D.C, p.237-268.
43. Brown DM, Bonte M, Bill R, Dawick J, Boogaard PJ (2017) Heavy hydrocarbon fate and transport in the environment. Quarterly J Eng Geol Hydrol 50:333-346.
44. Lu X, Zhang T, Fang HHP, Leung KMY. Zhang G (2011) Biodegradation of naphthalene by enriched marine denitrifying bacteria. Int Biodeterior Biodegrad 65:204–211.
45. Leahy JG, Colwell RR (1990) Microbial degradation of hydrocarbons in the environment. Microbiol. Rev 54: 305-315.
46. Pinyakong O, Habe H, Omori T (2003). The unique aromatic catabolic genes in sphingomonads degrading polycyclic aromatic hydrocarbons (PAHs). J Gen Appl Microbiol 49:1–19.
47. Greer CW, Whyte LG, Niederberger TD (2010) Microbial communities in hydrocarbon contaminated temperate, tropical alpine, and polar soils. *In* Timmis KN (ed.) Handbook of Hydrocarbon and Lipid Microbiology. Springer-Verlag, Berlin, p.2313-2328.
48. Zhang DC, Mortelmaier C, and Margesin R (2012) Characterization of the bacterial archaeal diversity in hydrocarbon-contaminated soil. Sci Total Environ 421-422:184-19.
49. Yang S, Wen X, Zhao L, Shi Y, Jin H (2014) Crude oil treatment leads to shirt of bacterial communities in soils from the deep active layer and upper permafrost along the China-Russia crude oil pipeline route. PLoS ONE 9(5):e96552.
50. Salam LB, Obayori OS, Nwaokorie FO, Suleiman A, Mustapha R (2017) Metagenomic insight into effects of spent oil perturbation on the microbial community composition and function in a tropical agricultural soil. Environ Sci Pollut Res DOI 10.1007/s11356-017-8364-3.
51. Auti AM, Narwade NP, Deshpande NM and Dhotre D (2019) Microbiome and imputed metagenome study of crude and refined petroleum-oil-contaminated soils: Potential for hydrocarbon degradation and plant-growth promotion. J Biosci 44:114 DOI: 10.1007/s12038-019-9936-9.
52. Wang Q, Wang C, Yu W, Turak A, Chen D, Huang Y, Ao J, Jiang Y, Huang Z (2018) Effect of nitrogen and phosphorus input on soil bacteria abundance, diversity and community composition in Chinese fir plantation. Front Microbiol Doi: 10:3389/fmicb.2018.01543.
53. Barka EA, Vatsa P, Sanchez L et al (2016) Taxonomy, physiology, and natural products of actinobacteria. Microbiol Mol Biol Rev 80:1–43.doi:10.1128/MMBR.00019-15.
54. Qin S, Li W- J, Dastager SG, Hozzein WN eds. (2016). Actinobacteria in Special and Extreme Habitats: Diversity, Function Roles and Environmental Adaptations. Lausanne: Frontiers Media. doi: 10.3389/978-2-88945-013-8.
55. Sutton NB, Maphosa F, Morillo JA, Al-Soud WA, Langenhoff AAM, Grotenhuis T, Rijnaart HHM, Smidt H (2013) Impact of long-term diesel contamination on soil microbial. Appl Environ Microbiol 79:619-630.
56. Williams KP, Sobral BW, Dickerman AW (2007) A robust species tree for the *Alphaproteobacteria*. J Bacteriol 189:4578-4586.
57. Williams KP, Gillespie JJ, Sobral BW, Nordberg EK, Snyder EE, Shallom JM, Dickerman AW (2010) Phylogeny of *Gammaproteobacteria*. J Bacteriol 192(9):2305-2314.
58. Borowik A, Wyszkowska J, Kucharski M, Kucharski J (2019) Implications of soil pollution with diesel oil and BP petroleum with ACTIVE technology for soil health Int J Environ Res Public Health 16:2474; doi:10.3390/ijerph1614247.
59. Hirano S, Kitauchi F, Haruki M, Imanaka T, Morikawa M, Kanaya S (2004). Isolation and characterization of *Xanthobacter polyaromaticivorans* sp. nov. 127W that degrades polycyclic and heterocyclic aromatic compounds under extremely low oxygen condition. Biosci Biotechnol Biochem 68(3): 557-564.
60. Teng Y, Wang X, Li L, Li Z, Luo Y (2015) Rhizobia and their bio-partners as novel drivers for functional remediation in contaminated soils. Front Plant Sci 6:32. doi: 10.3389/fpls.2015.00032.
61. Rochman FF, Sheremet A, Tamas I, Saidi-Mehrabad A, Kim J-J, Dong X, Sensen CW, Gieg LM, Dunfield PF (2017) Benzene and Naphthalene Degrading Bacterial Communities in an Oil Sands Tailings Pond. Front Microbiol 8:1845. doi: 10.3389/fmicb.2017.01845.
62. Pikuta E, Cleland D, Tang J **(**2003). Aerobic growth of *Anoxybacillus pushchinoensis* K1T: emended descriptions of *A. pushchinoensis* and the genus *Anoxybacillus.* Int J Syst Evol Microbiol 53:1561–1562.
63. Lee S-J, Lee Y-J, Ryu N, Park S, Jeong H, Lee S J, Kim B-C, Lee D-W, Lee H-S (2012) Draft genome sequence of the thermophilic bacterium *Anoxybacillus kamchatkensis* G10 J Bacteriol 6684:6684–6685.
64. Başbülbül G, Ozteber M, Bıyık HH, Oryaşın E, Bozdoğan B (2018) Presence of *entA* and *entB* genes among thermophilic bacteria isolated from hot springs in Turkey. Acta Microbiologica Bulgarica 34:1-7.
65. Margaryan A, Shahinyan G, Hovhannisyan P, Panosyan H, Birkeland N-K, Trchounian A (2018) *Geobacillus* and *Anoxybacillus* spp. from Terrestrial Geothermal Springs Worldwide: Diversity and Biotechnological Applications. D. Egamberdieva et al. (eds.), *Extremophiles in Eurasian Ecosystems: Ecology, Diversity, and Applications*, Microorganisms for Sustainability 8, <https://doi.org/10.1007/978-981-13-0329-6_5>.
66. Al-Jailawi MH, Mahdi MS, Fadhil AMA (2016) Investigation the consumption of aromatic hydrocarbons by *Anoxybacillus rupeinsis* Ir3 (JQ912241) using FTIR and HPLC analyses. British Biotechnol J 10(2):1-12.
67. De Boer AS, Priest F, Diderichsen B (1994) On the industrial use of *Bacillus licheniformis*: a review. Appl Microbiol Biotechnol 40:595–598.
68. Kolstø A-B, Tourasse NJ, Økstad OA (2009). What sets *Bacillus anthracis* apart from other *Bacillus* species? Annu Rev Microbiol 63:451–476.
69. Yuliani H, Sahlan M, Hermansya H, Wijanarko A (2012) Selection of polyaromatic hydrocarbon degrading bacteria. World Appl Sci 20(8):1133-1138.
70. Shivaji S, Chaturvedi P, Begum Z et al (2009) *Janibacter hoylei* sp. nov., *Bacillus isronensis* sp. nov. and *Bacillus* *aryabhattai* sp. nov., isolated from cryotubes used for collecting air from the upper atmosphere. Int J Syst Evol Microbiol 59:2977–2986.
71. Mual P, Singh NK, Verma A et al (2016) Reclassification of *Bacillus isronensis* Shivaji *et al.* 2009 as *Solibacillus isronensis* comb. nov. and emended description of genus *Solibacillus* Krishnamurthi *et al.* 2009. Int J Syst Evol 66:2113–2120.
72. Sielaff CA, Kumar RM, Pal D, Mayilraj S, Venkateswaran K (2017) *Solibacillus kalamii* sp. nov., isolated from a high-efficiency particulate arrestance filter system used in the International Space Station. Int J Syst Evol Microbiol 67:896–901. doi.org/10.1099/ijsem.0.001706.
73. Celikten H, Saral A, Kuzu SL (2019) Identifying bacterial communities in a full-scale woodchip biofilter. Pol J Environ Stud 28(5): 3655-3663.
74. Seuylemezian A, Singh NK, Vaishampayan P, Venkateswaran K (2017) Draft genome sequence of *Solibacillus kalamii*, isolated from an air filter aboard the International Space Station. Genome Announc 5:e00696-17. <https://doi.org/10.1128/genomeA> .00696-17.
75. Garrido-Sanz D, Manzano J, Martin M, Redondo-Nieto M, Rivilla R (2018) Metagenomic analysis of a biphenyl-degrading soil bacterial consortium reveals the metabolic roles of specific populations. Front Microbiol 9:232.
76. van Beilen J, Li Z, Duetz W, Smits T, Witholt B (2003) Diversity of alkane hydroxylase systems in the environment. Oil Gas Sci Technol. 58:427–440.
77. Hirano J, Miyamoto K, Ohta H (2005) Purification and characterization of the alcohol dehydrogenase with a broad substrate specificity originated from 2-phenylethanol-assimilating *Brevibacterium* sp. KU 1309. J Biosci Bioeng 100:318–322.
78. Zhou S, Zhang S, Lai D et al (2013) Biocatalytic characterization of a short-chain alcohol dehydrogenase with broad substrate specificity from thermophilic *Carboxydothermus hydrogenoformans*. Biotechnol Lett 35(3):359–336.
79. Garrido-Sanz D, Rodendo-Nieto M, Guirado M, Jimenez OP, Millan R, Martin M, Rivilla R (2019) Metagenomic insights into the bacterial functions of a diesel-degrading consortium for the rhizoremediation of diesel polluted soil. Genes 2019:10456, doi:10.3390/genes 100160456.
80. Harayama S, Kishira H, Kasai Y, Shutsubo K (1999) Petroleum biodegradation in marine environments. J Mol Microbiol Biotechnol 1:63–70.
81. Van der Meer JR, De Vos WM, Harayama S, Zehnder AJB (1992) Molecular mechanisms of genetic adaptation to xenobiotic compounds. Microbiol Rev 56: 677-694.
82. Resnick S, Lee K, Gibson D (1996) Diverse reactions catalyzed by naphthalene dioxygenase from *Pseudomonas* sp strain NCIB 9816. J Ind Microbiol 17:438–457.
83. Mouttaki H, Nanny MA, McInerney MJ (2007) Cyclohexane carboxylate and benzoate formation from crotonate in *Syntrophus aciditrophicus*. Appl Environ Microbiol 73:930–938.
84. Kung JW, Seifert J, von Bergen M, Boll M (2013) Cyclohexanecarboxyl-Coenzyme A (CoA) and cyclohex-1-ene-1- carboxyl-CoA dehydrogenases, two enzymes involved in the fermentation of benzoate and crotonate in *Syntrophus aciditrophicus.* J Bacteriol 195: 3193-3200.
85. Ang T-F, Maiangwa J, Salleh AB, Normi YM, Leow TC (2018) Dehalogenases: From Improved Performance to Potential Microbial Dehalogenation Applications. Molecules 23:1100; doi:10.3390/molecules23051100.
86. Temme HR, Carlson A, Novak PJ (2019) Presence, diversity, and enrichment of respiratory reductive dehalogenase and non-respiratory hydrolytic and oxidative dehalogenase genes in terrestrial environments. Front Microbiol 10:1258. doi: 10.3389/fmicb.2019.01258.
87. Janssen DB, Dinkla IJT, Poelarends GJ, Terpstra P (2005) Bacterial degradation of xenobiotic compounds: Evolution and distribution of novel enzyme activities. Environ Microbiol 7:1868–1882.
88. Koudelakova T, Chovancova, E, Brezovsky J, Monincova M, Fortova A, Jarkovsky J, Damborsky J (2011) Substrate specificity of haloalkane dehalogenases. Biochem J 435: 345–354.
89. Altenschmidt U, Oswal B, Fuchs G (1991) Isolation and characterization of benzoate-coenzyme A ligase and 2-aminobenxoate-coenzyme A ligase from a denitrifying *Pseudomonas* sp. J Bacteriol 173:5494-550.
90. Yin H, Wood TK, Smets BF (2005) Reductive transformation of TNT by *Escherichia coli*: pathway description. Appl Microbiol Biotechnol 67:397–404.
91. Saibu S, Adebusoye SA, Oyetibo GO (2020a) Aerobic bacterial transformation and biodegradation of dioxins: a review. Bioresour Bioprocess 7:7.
92. Saibu S, Adebusoye SA, Oyetibo GO, Rodriguez D (2020b) Aerobic degradation of dichlorinated dibenzo-p-dioxin and dichlorinated dibenzofuran by bacteria strains obtained from tropical contaminated soil. Biodegradation 31:123–137 (2020). <https://doi.org/10.1007/s10532-020-09898-8>.
93. Schomburg D and Salzmann M (1990) 2-Oxopent-4-enoate hydratase. In: Schomburg, D., Salzmann M. (eds) Enzyme Handbook 1. Springer, Berlin, Heidelberg.
94. Lee Y, Lee Y, Jeon CO (2019) Biodegradation of naphthalene, BTEX, and aliphatic hydrocarbons by *Paraburkholderia aromaticivorans* BN5 isolated from petroleum-contaminated soil. Sci Rep 9:860. <https://doi.org/10.1038/s41598-018-36165-x>.
95. Phale PS, Shah BA, Malhotra H (2019) Variability in assembly of degradation operons for naphthalene and its derivative, Carbaryl, Suggests mobilization through Horizontal gene transfer. *Genes* 10:569.
96. Pacwa-Płociniczak M, Płaza GA, Piotrowska-Seget Z, Cameotra SS (2011) Environmental applications of biosurfactants: recent advances. Int J Mol Sci 12(1):633‐654. doi:10.3390/ijms12010633.
97. Banat IM, Franzetti A, Gandolfi I, Bestetti G, Martinotti MG, Fracchia L, Smyth TJ, Marchant R (2010) Microbial biosurfactants production, applications and future potential. Appl Microbiol Biotechnol 87:427–444.
98. Saharan BS, Sahu RK, Sharma D (2011) A Review on Biosurfactants: Fermentation, current developments and perspectives. Genetic Eng Biotechnol J 2011:1–39.
99. Araújo SCdS, Silva-Portela RCB, de Lima DC et al (2020) MBSP1: a biosurfactant protein derived from a metagenomic library with activity in oil degradation. Sci Rep 10:1340 <https://doi.org/10.1038/s41598-020-58330-x>.
100. Nitschke M, Costa SG, Haddad R, Gon alves LA, Eberlin MN, Contiero J (2005) Oil wastes as unconventional substrates for rhamnolipid biosurfactant production by *Pseudomonas aeruginosa* B1. Biotechnol Prog 21:1562–1566.
101. Nwaguma IV, Chikere CB, Okpokwasili GC (2016). Isolation, characterization, and application of biosurfactant by *Klebsiella pneumoniae* strain IVN51 isolated from hydrocarbon‑polluted soil in Ogoniland, Nigeria. Bioresour Bioprocess 3:40.
102. Parales RE, Ju KS, Rollefson J, Ditty JL (2008) Bioavailability, transport and chemotaxis of organic pollutants. In: Diaz E (ed) Microbial Bioremediation. Caister Academic Press, Norfolk, pp 145–187.
103. Lai Q, Li W, Shao Z (2012) Complete genome sequence of *Alkanivorax dieselolei* type strain B5. J Bacteriol 194:6674.
104. Wang W, Shao Z (2013) Enzymes and genes involved in aerobic alkane degradation. Front Microbiol 4(116):1–7.

**Figure legends**

**Figure 1**: Comparative taxonomic profile of the FN1 and FN4 metagenomes at phylum level, computed by Kraken. Only phyla with significant biological differences as determined by STAMP (P < 0.05, difference between the proportions >1% and twofold of ratio between the proportions) are shown.

**Figure 2**: Comparative taxonomic profile of the FN1 and FN4 metagenomes at class delineation, computed by Kraken. Only classes with significant biological differences as determined by STAMP (P < 0.05, difference between the proportions >1% and twofold of ratio between the proportions) are shown.

**Figure 3**: Comparative taxonomic profile of the FN1 and FN4 metagenomes at genus delineation, computed by Kraken. All the genera detected in both metagenomes are shown. *Significant differences between FN1 and FN4 microcosms as determined by STAMP (P < 0.05, difference between the proportions >1% and twofold of ratio between the proportions, as determined by STAMP)

**Figure 4**: The benzoate degradation pathway performed using the KEGG pathway in KEGG Orthology. EC numbers in red are the benzoate degradation genes identified in FN1 microcosm. The identified genes include *pcaD*; 3-oxoadipate enol-lactonase [EC:3.1.1.24], mhpF; acetaldehyde dehydrogenase [EC:1.2.1.10], *aliB*; cyclohexanecarboxyl-CoA dehydrogenase [EC:1.3.99.-], pimeloyl-CoA dehydrogenase [EC:1.3.1.62], *benD-xylL*; dihydroxycyclohexadiene carboxylate dehydrogenase [EC:1.3.1.25 1.3.1.-], *benC-xylZ*; benzoate/toluate 1,2-dioxygenase reductase component [EC:1.18.1.-], *badH*; 2-hydroxycyclohexanecarboxyl-CoA dehydrogenase [EC:1.1.1.-], *had*; 6-hydroxycyclohex-1-ene-1-carbonyl-CoA dehydrogenase [EC:1.1.1.368], *dmpD*; 2-hydroxymuconate-semialdehyde hydrolase [EC:3.7.1.9], *ligC*; 2-hydroxy-4-carboxymuconate semialdehyde hemiacetal dehydrogenase [EC:1.1.1.312], *CMLE*; carboxy-cis,cis-muconate cyclase [EC:5.5.1.5], *pcaL*; 3-oxoadipate enol-lactonase / 4-carboxymuconolactone decarboxylase [EC:3.1.1.24 4.1.1.44], *acd*; glutaryl-CoA dehydrogenase (non-decarboxylating) [EC:1.3.99.32], cyclohex-1-ene-1-carbonyl-CoA dehydrogenase [EC:1.3.8.10], and cyclohexane-1-carbonyl-CoA dehydrogenase [EC:1.3.8.11]

**Figure 5**: The xylene degradation pathway performed using the KEGG pathway in KEGG Orthology. EC numbers in red are the xylene degradation genes identified in FN1 microcosm. The identified genes are *mhpF*; acetaldehyde dehydrogenase [EC:1.2.1.10], *benD-xylL*; dihydroxycyclohexadiene carboxylate dehydrogenase [EC:1.3.1.25 1.3.1.-], *benC-xylZ*; benzoate/toluate 1,2-dioxygenase reductase component [EC:1.18.1.-], *dmpD*; 2-hydroxymuconate-semialdehyde hydrolase [EC:3.7.1.9], *cymB*; p-cumic alcohol dehydrogenase, and *cmtB*; 2,3-dihydroxy-2,3-dihydro-p-cumate dehydrogenase [EC:1.3.1.58]
